# Comprehending Cardiac Dysfunction by Oxidative Stress: Untargeted Metabolomics of *In Vitro* Samples

**DOI:** 10.3389/fchem.2022.836478

**Published:** 2022-04-08

**Authors:** Alan Gonçalves Amaral, Isabela Aparecida Moretto, Flávia da Silva Zandonadi, Hans Rolando Zamora-Obando, Isabela Rocha, Alessandra Sussulini, André Alexandre de Thomaz, Regina Vincenzi Oliveira, Aline Mara dos Santos, Ana Valéria Colnaghi Simionato

**Affiliations:** ^1^ Laboratory of Analysis of Biomolecules Tiselius, Department of Analytical Chemistry, Institute of Chemistry, State University of Campinas, Campinas, Brazil; ^2^ Institute of Biology, Department of Structural and Functional Biology, State University of Campinas, Campinas, Brazil; ^3^ Laboratory of Bioanalytics and Integrated Omics, Department of Analytical Chemistry, Institute of Chemistry, State University of Campinas, Campinas, Brazil; ^4^ National Institute of Science and Technology for Bioanalytics—INCTBio, Institute of Chemistry, State University of Campinas, Campinas, Brazil; ^5^ Institute of Physics “Gleb Wataghin”, State University of Campinas, Campinas, Brazil; ^6^ Department of Chemistry, Federal University of São Carlos, São Carlos, Brazil

**Keywords:** metabolomics, cardiovascular diseases, oxidative stress, LC-MS, cell culture

## Abstract

Cardiovascular diseases (CVDs) are noncommunicable diseases known for their complex etiology and high mortality rate. Oxidative stress (OS), a condition in which the release of free radical exceeds endogenous antioxidant capacity, is pivotal in CVC, such as myocardial infarction, ischemia/reperfusion, and heart failure. Due to the lack of information about the implications of OS on cardiovascular conditions, several methodologies have been applied to investigate the causes and consequences, and to find new ways of diagnosis and treatment as well. In the present study, cardiac dysfunction was evaluated by analyzing cells’ alterations with untargeted metabolomics, after simulation of an oxidative stress condition using hydrogen peroxide (H_2_O_2_) in H9c2 myocytes. Optimizations of H_2_O_2_ concentration, cell exposure, and cell recovery times were performed through MTT assays. Intracellular metabolites were analyzed right after the oxidative stress (oxidative stress group) and after 48 h of cell recovery (recovery group) by ultra-high-performance liquid chromatography coupled to mass spectrometry (UHPLC-MS) in positive and negative ESI ionization mode. Significant alterations were found in pathways such as “alanine, aspartate and glutamate metabolism”, “glycolysis”, and “glutathione metabolism”, mostly with increased metabolites (upregulated). Furthermore, our results indicated that the LC-MS method is effective for studying metabolism in cardiomyocytes and generated excellent fit (R^2^Y > 0.987) and predictability (Q^2^ > 0.84) values.

## Introduction

Cardiovascular disease (CVD) refers to any sickness that affects the cardiovascular system and can be classified as heart, cerebral vascular, kidney, or peripheral arterial diseases. Their pathology is quite diverse, containing diseases such as cardiomyopathies, aneurysms, and others (Wilson et al., 1998; Kordalewska and Markuszewski, 2015). After the Second World War, such disorders became the leading cause of death in the world due to the lack of knowledge about the factors that would contribute to their onset, progression, and development. Nowadays, despite still being one of the main causes of death annually, studies like the Framingham Heart Study have shown that CVDs are associated with risk factors such as smoking, sedentary lifestyle, obesity, and diabetes (Wilson et al., 1998; Dórea and Lotufo, 2001; Lotufo, 2008).

The main risk factors of CVDs increase the generation of reactive oxygen species (ROS), which are normally produced by a natural process of cellular metabolism. ROS are composed of neutral species, such as hydrogen peroxide (H_2_O_2_) and hypochlorous acid (HClO), and radical species, such as the superoxide anion radical (O_2_·^−^). Although some of them are not very reactive, such as H_2_O_2_, they can react with other molecules or enzymes to form species and radicals with higher oxidizing strength (Hunter and Chien, 1999; Zuo et al., 2015; Panth et al., 20162016; Francisco et al., 2019). When these species are in excess in the body, due to an imbalance between them and antioxidant species, damage to important biological molecules occurs, resulting in tissue lesions and DNA damage. This condition is known as oxidative stress (Hermes-Lima et al., 1991; Meneghini, 1997; Rodrigo et al., 2013).

Several studies present the critical participation of ROS in the development of CVDs, such as higher production when therapeutic drugs (like doxorubicin) are administered (Ewer and Ewer, 2010; Clayton et al., 2020). However, the possible changes in metabolism and consequent effects on cardiac tissue have not been fully elucidated due to the complexity and diversity of molecular pathways. Such information is of paramount importance to find new markers and targets when devising new forms of diagnosis and treatments (Rebouças et al., 2016; Lee and Walsh, 2017; Tenreiro et al., 2021).

Within the methodologies currently used to explore the role of ROS in biological systems, metabolomics has gained considerable space as a powerful tool for understanding organisms at the molecular level (Zang et al., 2019). The goal of metabolomics is to study changes in the metabolome, which is composed of all biomolecules in a biological system of molecular mass up to 1,500 Da containing different chemical functions and variable concentration ranges found in a biological system (Fiehn, 2001; Barbosa et al., 2012; Viant et al., 2017). Several analytical platforms have been used for metabolomics analyses, such as gas chromatography–mass spectrometry (GC-MS) (Jonsson et al., 2004), liquid chromatography–mass spectrometry (LC-MS) (Cubbon et al., 2010), capillary electrophoresis–mass spectrometry (CE-MS) (Barbosa et al., 2013), and nuclear magnetic resonance (NMR) (Du et al., 2019), but LC-MS has been widely used for biological analysis and life science studies. Performing a metabolomics study in cellular culture (*in vitro* studies) avoids invasive techniques for obtaining biological samples. Moreover, *in vitro* systems have excellent advantages when compared with *in vivo* ones, such as the high metabolic homogeneity of the samples and its similarity to *in vivo* or *ex vivo* systems, which allows pre-test approaches to predict possible outcomes (Teng et al., 2009; Čuperlović-Culf et al., 2010; León et al., 2013).

The present study aims to perform an untargeted metabolomics investigation of differentiated cardiac cells of the H9c2 lineage, divided into three groups: healthy cells (control group), cells after simulated oxidative stress by H_2_O_2_ (oxidative stress group), and cells after recovery from oxidative stress (recovery group) by LC-MS. The purpose of this work is to identify changes in metabolism between each pair of groups (oxidative stress vs. control; recovery vs. control; and recovery vs. oxidative stress). Therefore, a thorough comprehension of these systems, and evaluation of the simulated oxidative stress on the metabolism may be achieved.

## Materials and Methods

### Chemicals and Materials

Ammonium formate (>99%), Dulbecco’s Modified Eagle’s Medium—high glucose (DMEM), sodium bicarbonate, Dulbecco’s Phosphate Buffered Saline (DPBS), retinoic acid (RA), 3-[4,5-dimethyl-2-thiazolyl]-2,5-diphenyl-2-tetrazolium bromide (MTT), and 2-propanol PA 99% were acquired from Sigma Chemicals (St Louis, MO, United States). Penicillin/streptomycin antibiotic, fetal bovine serum (FBS), trypsin-EDTA, primary antibody (anti-paxillin), secondary antibody (Alexa Fluor-546-conjugated goat anti-rabbit), Alexa Fluor-647 phalloidin, and Prolong with DAPI were acquired from ThermoFisher (Waltham, MA, EUA). Hydrogen peroxide PA was acquired from Fmaia (Belo Horizonte, MG, Brazil). Hydrochloric acid 36.5%–38.0% was acquired from J.T. Baker (Mexico). Methanol and acetonitrile (HPLC grade) were acquired from Merck (Darmstadt, HE, Germany), formic acid (LC-MS grade) was acquired from Fluka (Buchs, Switzerland), and water was purified in a Milli-Q system (Millipore, São Paulo, SP, Brazil). The internal standard leucine enkephalin acetate salt was acquired from Cayman Chemical.

### Cell Culture

H9c2 cell line, originally derived from ventricular myoblasts from rat embryos, was purchased from the American Type Culture Collection (ATCC). Cells were cultured in high-glucose DMEM supplemented with 3.7 g/L sodium bicarbonate, 10% FBS, and 1% penicillin/streptomycin solution at 37°C with 5% CO_2_ atmosphere in a humidified incubator. Medium was changed every 2 days, and cells were trypsinized (Trypsin-EDTA) and split as soon as they reached approximately 70% confluence. Cells were counted using a Neubauer chamber to determine the appropriate seeding densities for each experiment. Experiments were performed on cells between passages 28 and 31.

### Cell Differentiation

H9c2 cells were induced to cardiomyocyte differentiation through reduction of FBS content from 10 to 1% in the cell culture medium and addition of retinoic acid (RA) for 6 days. 10^6^ cells were plated and, once they reached approximately 80% confluence, differentiation was initiated by changing the medium to high-glucose DMEM (same supplementation) but with 1% FBS and 10 nmol/L retinoic acid (RA), resulting in an increase in cardiac-like cells. The differentiation medium was changed every 2 days (Pereira et al., 2011; Suhaeri et al., 2015; Patten et al., 2017).

### Cytotoxicity MTT Assay

In order to optimize the study parameters, two MTT assays were performed: (1) hydrogen peroxide (H_2_O_2_) concentration and exposure time to perform the oxidative stress; (2) cell recovery time. For the first assay, H9c2 undifferentiated cells were collected with trypsin, re-suspended, and seeded in 48-well multiplates. A total of 1.00 × 10^4^ cells were plated per well, in triplicates, as previously described. After the differentiation protocol, cells were treated with increasing concentrations of H_2_O_2_ (25, 50, 75, 100, 200, 400, and 600 μmol L^−1^) and different times of exposure (1, 4, 8, 12, and 24 h) to define the optimum dose and treatment time (Kim et al., 2014; Ilavenil et al., 2015; Zhang et al., 2018; Wu et al., 2019).

After treatment, the medium was removed, each well was washed with sterile DPBS, and 250 µl of MTT (1 g/L) solution was added to the cell monolayer. After 3 h of reaction in the incubator, the solution was removed and the formazan crystals were solubilized by adding 2-propanol solution acidified with HCl (0.1 mol/L). The plate was transferred to a spectrophotometer for absorbance determination at a wavelength of 550 nm in a microplate reader (BioTek SynergyTM 2 multimode). The mean optical density (OD) of 3 wells in each group was used to calculate cell viability as follows:

$$\mathrm{Cell Viability}=\;\frac{OD_{\mathrm{treatment}}-OD_{\mathrm{blank}}}{OD_{\mathrm{control}}-OD_{\mathrm{blank}}}$$

Addition of 500 μmol L^−1^ of H_2_O_2_ and 24 h of treatment were chosen for the next experiments.

For the second assay, a cell recovery test over 24 and 48 h was performed by replacing the culture medium containing H_2_O_2_ by a control one. Forty-eight hours of recovery was selected for this study.

### Morphological Assessment

Undifferentiated H9c2 cells exhibit a fusiform morphology, i.e., elongated, with the ends narrower than the center, and mononucleated (Kimes and Brandt, 1976). After the differentiation process, their morphology becomes long with fine branching cardiomyocytes, and multinucleate. To follow the morphological change, an inverted microscope was used before differentiation and on days 2, 4, and 6 of differentiation. Changes after exposure to hydrogen peroxide were also captured after 24 h of oxidative stress, as well as at 24 and 48 h of recovery after oxidative stress (Pereira et al., 2011).

### Immunofluorescence Staining and Microscopy Analysis

H9c2 cells were fixed with 4% paraformaldehyde, blocked, and permeabilized with 3% bovine serum albumin and 0.1% Triton-X in 0.1 mol L^−1^ DPBS on ice. Then, cells were incubated with anti-paxillin (1:200) or with anti-γH2AX (1:200) primary antibodies for 30 min at room temperature. Next, cells were labeled with Alexa Fluor-546-conjugated goat anti-rabbit (1:2,000) secondary antibody, and Alexa Fluor-647 phalloidin for 30 min at room temperature. Slides were then mounted with Prolong with 4′,6-diamidino-2-phenylindole (DAPI). Fluorescence images were acquired on a Zeiss Elyra PS.1 microscope. “Panoramic” images were examined by an EC Plan-Neofluar 10x/0.30 objective in the laser WideField mode. “Zoomed in” images were collected by a Plan-Apochromat 63x/1.4 Oil DIC objective in the 3D-SIM mode. 3D-SIM z stacks were projected on a single plane with summed intensities using the ImageJ-FIJI software.

### Sample Preparation

Samples were divided into three groups: 3 samples of healthy cells (control group), 3 samples of cells that underwent oxidative stress with 500 μmol L^−1^ H_2_O_2_ for 24 h (oxidative stress group), and 3 samples of cells that recovered during 48 h from oxidative stress (recovery group). For cell metabolomics, the cardiomyoblasts were lightly scraped with a rubber cell scraper, and suspended in 800 µl of ice-cold methanol (MeOH) extraction solvent. Samples were subjected to three freeze–thaw cycles for complete cell disruption. For this purpose, samples were placed in liquid nitrogen for 10 min for rapid freezing and thawed in an ice bath for 10 min. The supernatant was recovered by centrifugation at 5,725 × *g* for 5 min at 4°C; the cell pellet was extracted once with 400 µl of MeOH and combined with the previous supernatant. Samples were stored at −80°C until analysis. For quality control samples (QCs), 200 µl from each of the samples was aliquoted, pooled, and separated into equal volumes (Dettmer et al., 2011; Mastrangelo et al., 2016).

### LC-MS Analysis

RPLC-MS and HILIC-MS analysis were conducted on an Agilent UHPLC system (model 1,260 Infinity II, Agilent Technologies, California, EUA). Separations were performed on an Ascentis Express C18 column (2.7 µm, 150 mm × 2.1 mm, Supelco Inc., Missouri, EUA) for RPLC, an Atlantis Silica column (1.7 µm, 100 mm × 2.1 mm, Waters, Massachusetts, EUA) for HILIC in positive ionization mode, and an ACQUITY BEH Amide column (1.7 µm, 100 mm × 2.1 mm, Waters, Massachusetts, EUA) for HILIC in negative ionization mode, both kept at 15°C during analyses. For RPLC in both ionization modes and HILIC in negative ionization mode, the mobile phase was composed of the following: A—water + 0.1% formic acid, and B—acetonitrile + 0.1% formic acid. For HILIC in positive ionization mode, the mobile phase was composed of the following: A—ammonium formate 100 mmol/L, and B—acetonitrile + 0.1% formic acid. Injection volumes of 2.0 µl and a flow rate of 400 μL/min were used. The gradients applied in the two separation modes are shown in Table 1.

**TABLE 1:** Elution gradient of the chromatographic methods. B: Acetonitrile + 0.1% formic acid.

RPLC-MS (ESI+) and (ESI-)		HILIC-MS (ESI+) and (ESI-)	
Time (min)	B (%)	Time (min)	B (%)
0	1	0	99
3	2	3	98
10	20	10	70
15	60	15	40
18	85	18	15
20	90	20	10
25	95	20,1	1
30	95	22	1
31	99	22,1	99
33	99	25	99
33,1	1		
35	1		

Mass spectra were acquired using an Impact HD QTOF™ (Bruker Daltonics, Bremen, Germany) mass spectrometer with ESI ionization. The MS was operated in positive and negative ESI modes. The following parameters were used to register mass spectra: capillary voltage: 3600 V; end plate offset: 450 V; nebulizer gas: 4.0 bar; drying gas flow rate: 8.0 L/min; drying gas temperature: 180°C; collision cell energy: 5.0 eV; MS range (full scan): 50.0–1,300.0 Da.

In addition, 3 μg/ml leucine enkephalin acetate salt (C_28_H_37_N_5_O_7_) was used as internal standard for RPLC and HILIC, presenting exact mass of 556.2765 Da ([M + H]^+^) in positive ionization mode, and 554.2609 Da ([M-H]^−^) in negative ionization mode. The mass spectrometer was programmed to perform data acquisition in data-dependent acquisition (DDA) mode.

### Data Processing

Raw data files were calibrated automatically by application of DataAnalysis 4.2 (Bruker Daltonics, Bremen, Germany). The mzXML files were pre-processed by application of MZmine 2.53 (Pluskal et al., 2010), and the parameters present in Supplementary Table S1 have been applied for MS and MS/MS scans.

### Statistical Analysis

The resulting data matrix has been exported as a .csv file. Features with at least 30% of RSD of the QCs, peak detection ratio less than 70%, and/or at least 5% of blank contribution were removed. Further data pre-processing was performed with MetaboAnalyst 5.0 (Pang et al., 2021), using Log transformation and auto scaling normalization for RPLC-ESI(+)-MS, RPLC-ESI(-)-MS, and HILIC-ESI(+)-MS, while auto scaling was used on HILIC-ESI(-)-MS. Multivariate analyses (PCA and PLS-DA) were applied to check trends, VIP scores indicated significant features for group separation, while unpaired *t*-test with false discovery rate (FDR) showed which of the features were statistically significant. From the PLS-DA model, a cross-validation was performed to evaluate the R^2^Y (goodness of fit) and Q^2^ (goodness of prediction) values, and a permutation test was carried out (2000 permutations).

### Metabolite Annotation and Pathway Analysis

Statistically significant metabolites, i.e., FDR < 0.05, *p* value < 0.05 and VIP score > 1.0, were searched in the CEU Mass Mediator (Gil-de-la-Fuente et al., 2019) tool in order to perform a tentative identification using their *m/z* values from MS1 spectra. Kyoto Encyclopedia of Genes and Genome (KEGG) (Kanehisa et al., 2019), Human Metabolome Database (HMDB) (Wishart et al., 2018), LipidMaps (www.lipidmaps.org), and PubChem (Kim et al., 2021) public databases were used for identification with a maximum error of 10 ppm. For the possible adducts, [M + H]^+^, [M + Na]^+^, [M + K]^+^, [M + NH_4_]^+^, [2M + H]^+^, [M+2H]^2+^, [M+2Na]^2+^, [M+2K]^2+^, [M + H + Na]^2+^, [M + H + K]^2+^, [M + H + NH_4_]^2+^, [M+2Na-H]^+^, [M+2K-H]^+^, [M + H-H_2_O]^+^, and [M + H-2H_2_O]^+^ were considered for positively charged adducts, while [M-H]^-^, [M + Cl]^-^, [M-H-H_2_O]^−^, [2M-H]^−^, [M-2H]^2−^, [M + Na-2H]^−^, [M + K-2H]^-^, and [M + FA-H]^−^ were considered for negatively charged ones. In an attempt to validate the annotated features using the MS^2^ spectra acquired by DDA, a comparison was performed with literature MS^2^ spectra available in the PubChem chemical information library (Kim et al., 2021). MetaboAnalyst 5.0 Pathway Analysis tool provided the impact on metabolic pathways, and the possible connections of these changes with oxidative stress-related CVDs.

## Results

### Cytotoxicity MTT Assay

In order to obtain information regarding cell viability against H_2_O_2_ treatment, MTT assays were performed. Our data showed a general decrease in cell viability with the increment of H_2_O_2_ concentration after 12 and 24 h of exposure (Supplementary Figure S1A), although 4 and 8 h resulted in an increase in cell viability that reached higher values than the control samples. Myocytes exposed to H_2_O_2_ for 24 h presented a gradual reduction in cell viability, reaching approximately 50% when treated with 400 and 600 μmol/L of H_2_O_2_. Thus, 24 h was chosen as the optimal time of cell exposure for the proposed experimental design. Since cell viability was similar for 400 and 600 μmol/L of H_2_O_2_, according to standard deviations (0.544 ± 0.015 for the former, and 0.471 ± 0.059 for the latter), 500 μmol/L intermediate value was used in the present study.

The recovery time assay showed that after 24 h of recovery, there was 80% reduction in the cardiomyocyte viability. A possible reactivation of cell proliferation was also noted after 48 h recovery time, since the viability value reached the same value of the control (Supplementary Figure S1B).

### Morphological Assessment and Immunofluorescence Staining

**FIGURE 1:**
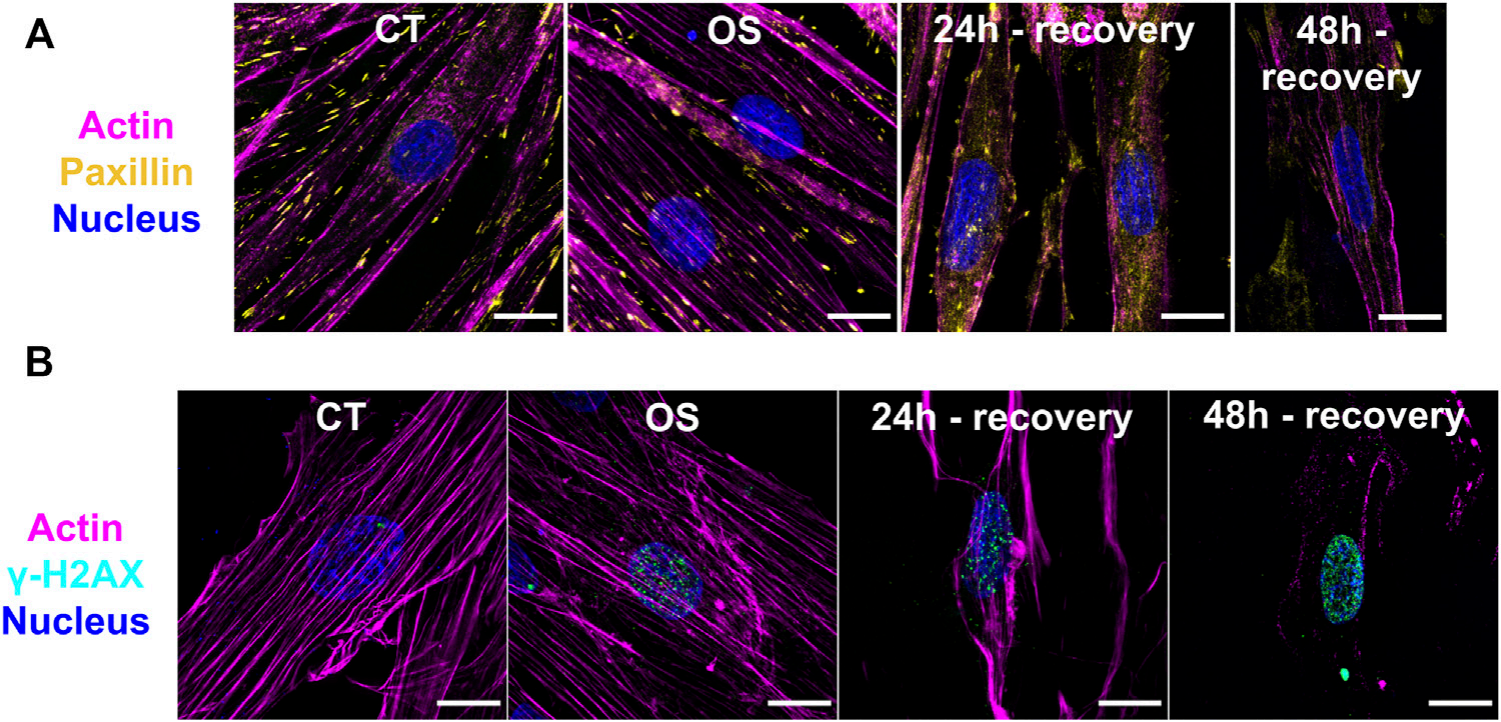
Immunofluorescence images from H9c2 cardiomyocytes after oxidative stress and different recovery times. **(A,B)** SR-SIM microscopy of cardiomyocytes from Control (CT), oxidative stress (OS), and 24- and 48-h recovery groups. Magenta: actin; Yellow: paxillin; Cyan: γH2Ax; Blue: nucleus. Scale bar = 15 µm.

The differentiated cardiomyocytes were morphologically elongated and multinucleated, while the undifferentiated cells showed a polygonal shape (Supplementary Figure S2A). Morphological damages were observed in the oxidative stress group and in cells after recovery for 24 and 48 h (Supplementary Figure S2B). Triple-labeled fluorescence super-resolution microscopy showed control cells with an extensive cytoplasm, preserved actin filaments, and focal adhesions sites (Figure 1A, Supplementary Figure S3). Few DNA damage sites were confirmed in control cells by the low number of γH2AX foci (Figure 1B). Cells submitted to oxidative stress showed a similar morphology; however, a substantial increase of DNA damage sites were observed, as expected (Figure 1B). On the other hand, myocytes recovered for 24 h presented cytoplasm retraction and disassembly of the cytoskeleton accompanied by large DNA damage increase. Cells recovered for 48 h of oxidative stress had a smaller number of focal adhesions, cytoplasm retraction, paxillin accumulation on the cytosol, and extensive DNA damage (Figures 1A,B). Paxillin was also accumulated on distinct points of the extracellular matrix, probably coming from cell debris. These findings indicate that the generation of ROS had a negative effect in the H9c2 cardiomyocyte architecture and metabolism.

### LC-MS Analysis

**FIGURE 2:**
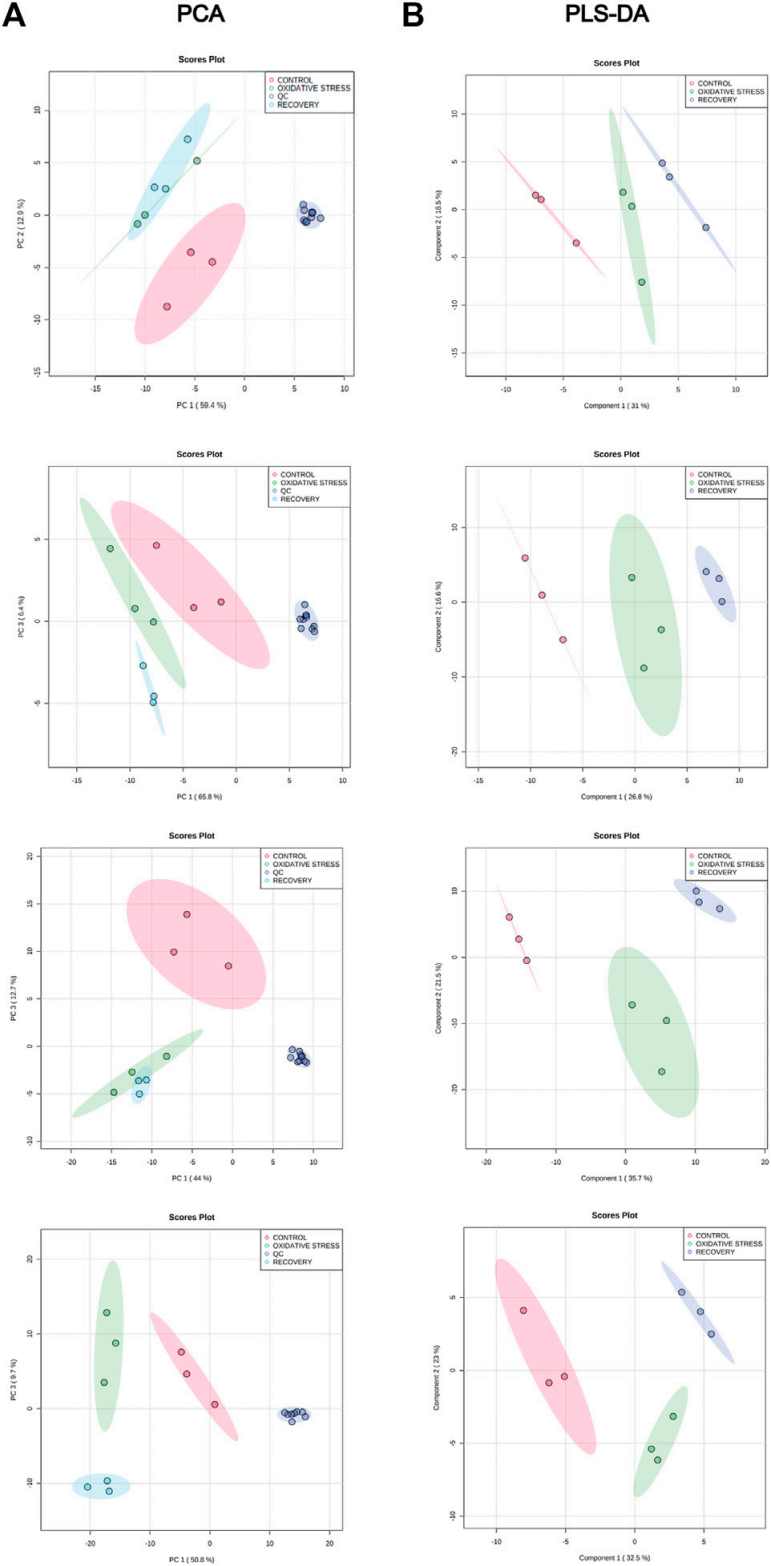
**(A)** PCA for control (red), oxidative stress (green), recovery (light blue), and QCs (blue) samples for RPLC-ESI(+)-MS, RPLC-ESI(−)-MS, HILIC-ESI(+)-MS, and HILIC-ESI(−)-MS modes, from top to bottom. **(B)** PLS-DA score plot for control (red), oxidative stress (green), and recovery (blue) samples for RPLC-ESI(+)-MS, RPLC-ESI(−)-MS, HILIC-ESI(+)-MS, and HILIC-ESI(−)-MS modes, from top to bottom.

The analysis of the intracellular metabolome was performed after completion of the cell growth and stress stages. The data were processed by MZmine software version 2.53 resulting in 704 (RPLC-ESI(+)-MS), 327 (RPLC-ESI(-)-MS), 427 (HILIC-ESI(+)-MS), and 811 (HILIC-ESI(-)-MS) molecular features. After data filtering with RSDs of QC samples and blank contribution, 101 (RPLC-ESI(+)-MS), 85 (RPLC-ESI(-)-MS), 198 (HILIC-ESI(+)-MS), and 430 (HILIC-ESI(-)-MS) features remained. Principal component analysis (PCA) was used to check data integrity (Figure 2A), showing a good pattern of grouping among the samples and clustered QCs, which indicates the instrumental variation was lower than the biological one.

PLS-DA models were also built without the QC samples to maximize the discrimination between groups since it is a supervised method (Figure 2B). Cross-validation results shows the performance parameters, R^2^Y and Q^2^ (Table 2), indicating that the models had an excellent goodness of fit (R^2^Y ≈ 1) and a good prediction (Q^2^ > 0.84). The statistical significance of Q^2^ was evaluated by means of a permutation test using 2,000 random permutations. The *p*-values <0.05 show that Q^2^ is statistically significant (Table 2).

**TABLE 2:** R^2^Y, Q^2^, and permutation (*p*) values for the evaluation of statistical significance.

	R^2^Y	Q^2^Y	Permutation test (*p*)
RPLC-ESI(+)-MS	0.9915	0.8405	<5 × 10^–4^
RPLC-ESI(−)-MS	0.9952	0.8428	<5 × 10^–4^
HILIC-ESI(+)-MS	0.9871	0.8847	0.011
HILIC-ESI(−)-MS	0.9995	0.8855	0.0335

### Metabolite Annotation and Pathway Analysis

**FIGURE 3:**
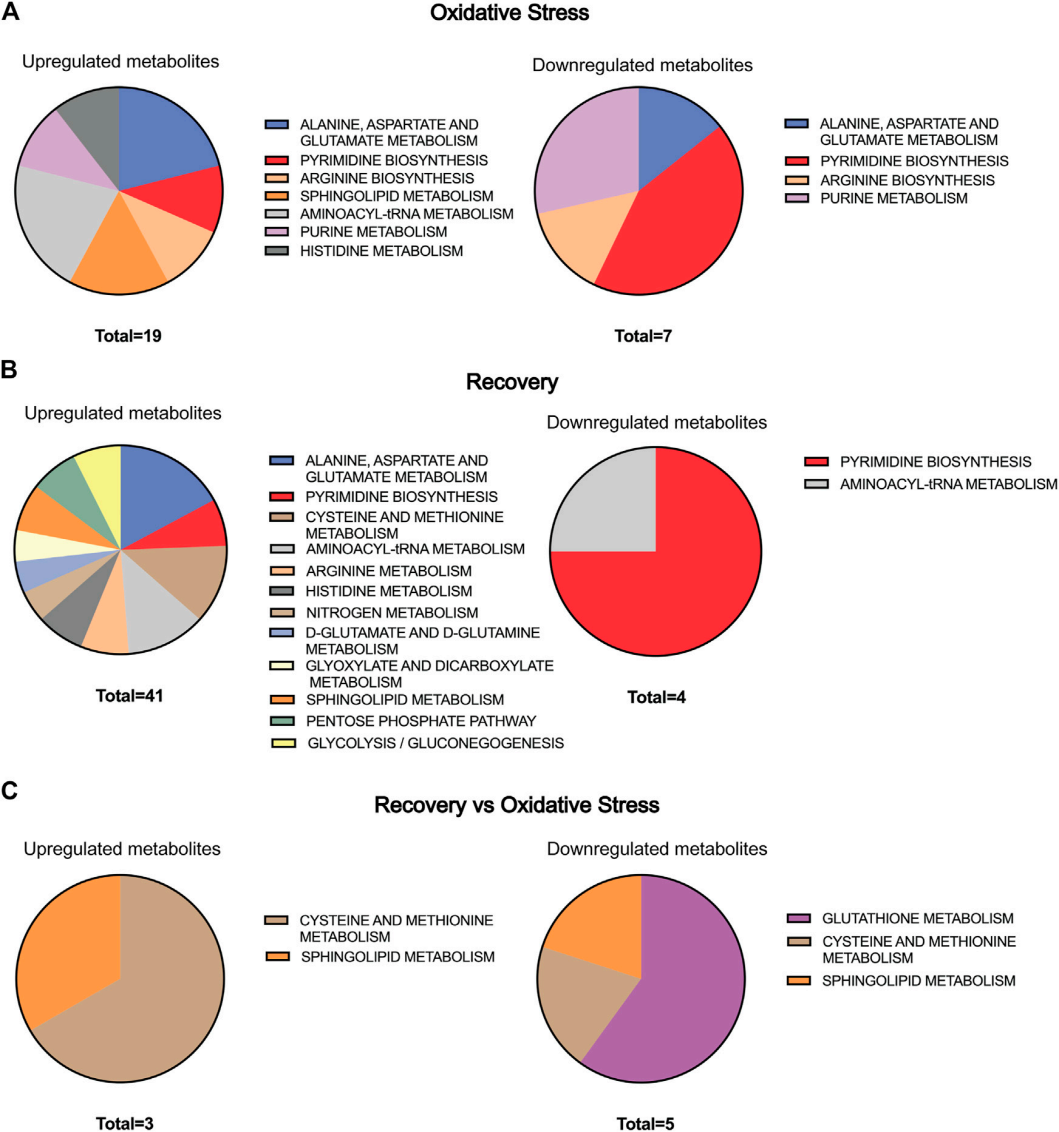
Pie chart representation of the statistically significant metabolic pathway present in Table 3 for **(A)** oxidative stress, **(B)** recovery, and **(C)** recovery vs. oxidative stress. The size of the pie chart is represented by the number of up- and downregulated metabolites in each group.

To perform biological interpretation of the results obtained from LC-MS analyses, metabolite annotation was performed. Thirty-four metabolites from the OS group were compared to the control one, while 69 metabolites from the 48-h recovery group were used for the same comparison (Supplementary Tables S2, S3). Twenty-three metabolites in recovery vs. OS group (Supplementary Table S4) were significantly altered. Pathway Analysis tool of MetaboAnalyst 5.0 revealed 7 pathways significantly altered (*p* value < 0.05) in OS, 12 in recovery, and 3 in recovery vs. OS (Table 3 and Supplementary Figure S4). Next, the up- and downregulated metabolites of the major pathways were compared using pie charts (Figure 3). In the OS group, 19 metabolites were upregulated and 7 were downregulated, within the pathways “alanine, aspartate and glutamate metabolism”, “pyrimidine biosynthesis”, “arginine biosynthesis”, “arginine biosynthesis”, and “purine biosynthesis”. Moreover, only upregulated metabolites were identified in the pathways “sphingolipid metabolism”, “aminoacyl-tRNA metabolism”, and “histidine metabolism” in the cells exposed to hydrogen peroxide.

**TABLE 3:** Statistically significant metabolic pathways (*p* value < 0.05) altered in H9c2 cardiomyocytes upon H_2_O_2_-induced oxidative stress. *p value < 0.01.

Pathways (OS vs. Control)	*p* value	FDR	Impact	Metabolites up	Metabolites down
Alanine, aspartate, and glutamate metabolism	6.7111·10^–5^	0.0056	0.4070	4 (21.05%)	1 (14.29%)
Pyrimidine metabolism	3.4662·10^–4^	0.0146	0.0912	2 (10.53%)	3 (42.86%)
Arginine biosynthesis	0.0014	0.0378	0.1168	2 (10.53%)	1 (14.29%)
Sphingolipid metabolism	0.0046	0.0958	0.0284	3 (15.79%)	0 (0.00%)
Aminoacyl-tRNA biosynthesis	0.0073	0.1232	0.1667	4 (21.05%)	0 (0.00%)
Purine metabolism	0.0210	0.2940	0.0230	2 (10.53%)	2 (28.57%)
Histidine metabolism	0.0281	0.3373	0.0902	2 (10.53%)	0 (0.00%)
**Pathways (Recovery vs. Control)**	***p* value**	**FDR**	**Impact**	**Metabolites Up**	**Metabolites Down**
Alanine, aspartate and glutamate metabolism	1.915·10^–5^	0.0016	0.6691	7 (17.07%)	0 (0.00%)
Pyrimidine metabolism	0.0013	0.0561	0.0963	3 (7.32%)	3 (75.00%)
Cysteine and methionine metabolism	0.0037	0.0841	0.1220	5 (12.20%)	0 (0.00%)
Aminoacyl-tRNA biosynthesis	0.0040	0.0841	0.1667	5 (12.20%)	1 (25.00%)
Arginine biosynthesis	0.0095	0.1536	0.1168	3 (7.32%)	0 (0.00%)
Histidine metabolism	0.0139	0.1536	0.0492	3 (7.32%)	0 (0.00%)
Nitrogen metabolism	0.0146	0.1536	0.0	2 (4.88%)	0 (0.00%)
D-Glutamine and D-glutamate metabolism	0.0146	0.1536	0.5	2 (4.88%)	0 (0.00%)
Glyoxylate and dicarboxylate metabolism	0.0189	0.1767	0.0741	2 (4.88%)	0 (0.00%)
Sphingolipid metabolism	0.0294	0.2469	0.0284	3 (7.32%)	0 (0.00%)
Pentose phosphate pathway	0.0385	0.2942	0.1126	3 (7.32%)	0 (0.00%)
Glycolysis/Gluconeogenesis*	0.0590	0.4131	0.0425	3 (7.32%)	0 (0.00%)
**Pathways (Recovery vs. OS)**	***p* value**	**FDR**	**Impact**	**Metabolites Up**	**Metabolites Down**
Glutathione metabolism	0.0036	0.2425	0.3199	0 (0.00%)	3 (60.00%)
Cysteine and methionine metabolism	0.0058	0.2425	0.0221	2 (66.67%)	1 (20.00%)
Sphingolipid metabolism	0.0235	0.6575	0.0243	1 (33.33%)	1 (20.00%)

Interestingly, 35 metabolites were upregulated and 3 were downregulated after 48 h of cell recovery, with only downregulated metabolites found in “pyrimidine biosynthesis” and “aminoacyl-tRNA metabolism”. Among the upregulated metabolites, “alanine, aspartate and glutamate metabolism” stands out, with 7 altered metabolites. Furthermore, comparisons of pathways related to oxidative stress and recovery after 48-h groups, despite not indicating a large number of alterations, pointed to “glutathione metabolism”, an important pathway for intracellular antioxidant activity and for maintaining cellular homeostasis.

**FIGURE 4:**
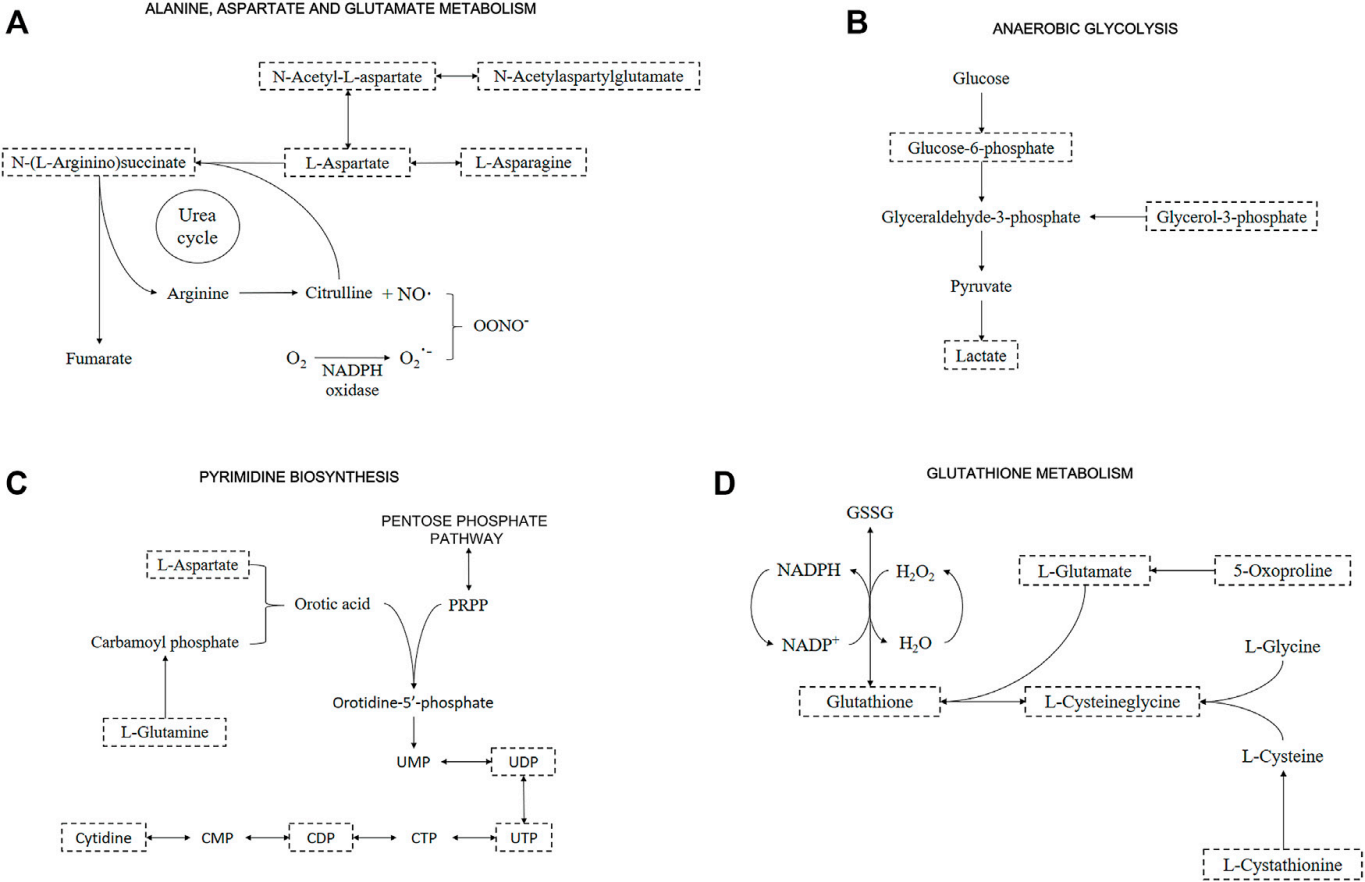
Representative metabolic pathway maps of significantly altered metabolites. Boxes with dashed lines represent the identified metabolites. Important metabolites related to **(A)** “Alanine, aspartate and glutamate metabolism” and “Urea cycle”; **(B)** “Anaerobic glycolysis”; **(C)** “Pyrimidine biosynthesis”; and **(D)** “Glutathione metabolism”.

Metabolites of “alanine, aspartate and glutamate metabolism” were upregulated (i.e., L-Aspartate, L-Asparagine, L-Glutamate, L-Glutamine, N-Acetyl-L-aspartate, and N-Acetylaspartylglutamate) in both OS and after recovery for 48-h groups (Figure 4A and Supplementary Figure S5A). The only downregulated metabolite of this pathway was N-(L-Arginine)succinate in the OS group, being an intermediate of the “arginine biosynthesis” and “urea cycle” (Figure 4A). Several studies on this pathway show a connection with oxidative stress due to the intracellular production of nitric oxide (NO) (Rochette et al., 2013).

A pathway identified by its significant metabolites is “anaerobic glycolysis”, represented by increased Glucose-6-phosphate, Glycerol-3-phosphate, and Lactate under all conditions (Figure 4B and Supplementary Figure S5B). This pathway stands out for energy production in the form of ATP inside the cells, but in oxidative stress situations, species such as Lactate promote cellular defense mechanisms (Tauffenberger et al., 2019). By altering Glucose-6-phosphate and Lactate, alterations in “pentose phosphate pathway” are detected, supported by the presence of Gluconate-6-phosphate downregulated in the OS group. This pathway is directly related to “pyrimidine biosynthesis” and “purine biosynthesis” due to the formation of phosphoribosyl pyrophosphate (PRPP), an important metabolite in both pathways. Although PRPP was not altered, Uridine 5′-diphosphate (UDP), Uridine 5′-triphosphate (UTP), and Cytidine 5′-diphosphate (CDP) were downregulated, and Cytidine was upregulated in OS and recovery groups (Figure 4C and Supplementary Figure S5C).

Glutathione levels were upregulated in the control group when compared to the recovery one. On the other hand, the OS group showed a downregulation pattern when compared to the recovery one. Among other altered metabolites from “glutathione metabolism”, L-Cysteinylglycine and 5-Oxoproline were downregulated, while L-Cystathionine was upregulated in recovery vs. OS groups (Figure 4D and Supplementary Figure S5D). Additionally, other relevant metabolites showed significant changes, although they did not necessarily appear in significant pathways. Sphingosine 1-phosphate and the metabolism it participates in, “sphingolipid metabolism”, were significant in all conditions, presenting upregulated. Additionally, carnosine was upregulated in OS; taurine was slightly upregulated and adenosine 5′-monophosphate (AMP) was downregulated in the recovery group; carnitine was upregulated and All-trans-4-oxoretinoic acid was downregulated in recovery vs. OS group (Supplementary Figure S5E). Afterwards, features validation was performed by obtaining the MS^2^ spectra, and the following metabolites were identified: L-Aspartate, L-Asparagine, Glycerol 3-phosphate, CDP, L-Cystathionine, Taurine, AMP, L-Carnosine, and L-Carnitine (Supplementary Figure S6−13).

## Discussion

Oxidative stress (OS) has shown to play an important role in the pathophysiology of CVDs. A balance between the levels of reactive oxygen species (ROS) and the antioxidant defenses is essential for cell health (Rodrigo et al., 2013; van der Pol et al., 2019; Alkadi, 2020). Moderated concentration of ROS has important roles in cell signaling, whereas excessive levels of ROS cause damage to macromolecules such as protein, lipids and DNA, which can trigger cell death (Meneghini, 1997; Poljsak et al., 2013; Alkadi, 2020). Although several signaling pathway studies and functional *in vivo* experiments have demonstrated the effects of ROS production in CVD, scarce information is available about measurable changes in the metabolomic profile of cardiomyocytes undergoing oxidative stress. In the current study, PCA and PLS-DA analyses allowed identification of the predominant metabolites and pathways modulated by OS in cardiomyocytes H9c2.

Our results showed that a key pathway to be activated under oxidative stress was the alanine, aspartate, and glutamate metabolism. Specifically, we found that the L-aspartate and L-Asparagine were upregulated 2.5- and 1.56-fold, respectively, while the N-(L-Arginino)succinate was downregulated 1.76-fold in cardiomyocytes exposed to H_2_O_2_ over control cells (Figure 4A), probably due to increased utilization or decreased synthesis. Although we cannot firmly distinguish between these possibilities, it is likely that N-(L-Arginine)succinate is depleted because of increased utilization. L-aspartate is converted into N-(L-Arginine)succinate, which may be converted into L-arginine, the substrate of nitric oxide (NO) synthase. NO is a potent anti-hypertensive agent, produced when L-arginine is converted to citrulline (Hou et al., 2017). Under oxidative stress, the increased ROS concentrations reduce the amount of bioactive NO by chemical inactivation to form the peroxynitrite anion (ONOO^−^), resulting in both oxidation and nitration of proteins and in lipid peroxidation (Toyokuni, 2008; Rochette et al., 2013). This pathway was also upregulated in the cardiomyocytes recovered for 48 h after oxidative stress.

The observed upregulation of the “aspartate metabolism” under OS may also affect related pathways including “glycolysis”, “gluconeogenesis”, “citric acid cycle”, “amino acids biosynthesis”, and “pyrimidine and purine biosynthesis” (Bielarczyk et al., 1986; Darvey, 2000; Weckmann et al., 2018). Interestingly, upon 48-h recovery of OS, we observed an upregulation of the glycolysis metabolites such as Glucose-6-phosphate (2.01-fold), Glycerol-3-phosphate (0.8-fold), and Lactate (1.59-fold) (Figure 4B), which indicated that the energy metabolism shifts to anaerobic glycolysis as an adaptive response to oxidative stress in cardiomyocytes H9c2. We also identified a modest accumulation in citrate levels (0.48-fold) and an upregulation of amino acid biosynthesis pathways (Figure 3), indicating a dysfunction of the citric acid cycle and a shift towards amino acids biosynthesis (Mullarky et al., 2015). Scientific literature have reported the action of the factor HIF-1α during oxidative stress in mediation of gene expression involved in shifting the metabolism towards anaerobic glycolysis without requirement of a hypoxic condition (Shi et al., 2009; Frezza et al., 2011). HIF-1α upregulates the expression of Glut1 and glycolytic enzymes increasing the anaerobic glycolysis pathway (Fulda and Debatin, 2007). Moreover, HIF-1α has shown to limit the conversion of pyruvic acid to acetyl-CoA in the mitochondria, impairing the citric acid cycle activity as a strategy to couple to oxidative stress (Kim et al., 2006; Papandreou et al., 2006; Weckmann et al., 2018).

The metabolite L-carnitine was also upregulated (1.9-fold) in cardiomyocytes under oxidative stress. This metabolite facilitates the long-chain fatty acids transport into the mitochondrial matrix in order to be degraded by beta oxidation. The fatty acids conjugated to coenzyme A are transferred to carnitine by the enzyme carnitine acyltransferase I, resulting in the production of fatty acid acyl-carnitine. This metabolite is transported across the inner mitochondrial membrane *via* carnitine translocase, which exchanges long-chain acyl-carnitine for carnitine. Inside the mitochondria, the acyl group from fatty acyl-carnitine is transferred to coenzyme-A by carnitine acyltransferase II. Then, the long-chain acyl-CoA enters beta-oxidation pathway, which results in the production of acetyl-CoA, a substrate for citric acid cycle. Studies suggest that L-carnitine protects cardiomyocytes from oxidative stress and toxic myocardial injury, such as by doxorubicin exposure (Wang et al., 2018). In this case, upregulation of L-carnitine may be related to a reduction in intra-mitochondrial acetyl-CoA in response to the mitochondria oxidative damage, leading to stimulation of pyruvate dehydrogenase, enhancing anaerobic glycolysis, and reducing beta oxidation (Mate et al., 2010).

We also demonstrated a regulation of the “pyrimidine and purine biosynthesis” in cardiomyocytes under oxidative stress (Figure 4C). Interestingly, although the upstream metabolites L-glutamine (1.56-fold), L-aspartate (2.5-fold), and cytidine (1.86-fold) were upregulated, the products UDP, CDP, and AMP of the pyrimidine and purine metabolism were strongly downregulated. The metabolites UDP and CDP were downregulated 3.28- and 3.49-fold in the OS group and 2.47- and 4.5-fold in the recovery group, respectively. We also identified a downregulation in the AMP levels (3.61-fold) in the recovery group. The downregulation of AMP without alterations in the ATP levels increases the ATP/AMP ratio and inhibits AMPK, the main sensor of cellular energy status in eukaryotic cells (Garcia and Shaw, 2017; Lin and Hardie, 2018). AMPK inhibits anabolic pathways to avoid wasting energy through the inhibition of mTORC1, under stressful conditions. The kinase mTORC1 regulates key cellular functions, which promotes cell growth and survival (Hardie, 2014; Saxton and Sabatini, 2017; González et al., 2020; Ling et al., 2020). In general, our results suggest that oxidative stress induces a metabolic adaptation mechanism that allows cell survival and the reactivation of the anabolic pathways in cardiomyocytes recovered from the oxidative stress condition.

A significant alteration of glutathione occurred after cardiomyocytes recovered from oxidative stress. The main function of glutathione is to avoid oxidative stress, reducing the deleterious effects of ROS on the cardiomyocytes, constituting the most abundant cellular antioxidant (Matuz-Mares et al., 2021). Glutathione acts on redox equilibrium on its reduced form, trapping ROS, and preventing the inactivation of compounds such as NO (Ceconi et al., 2000; Guerra, 2001; Pompella et al., 2003; Matuz-Mares et al., 2021). Within glutathione consumption, it is easily replaced if the amino acids L-glutamate, L-cysteine, and L-glycine are available, as provided by DMEM medium. Usually, cysteine is found in lower amounts and is one of the limiting factors for glutathione synthesis. In such cases, dipeptides such as cystathionine (1.35-fold) can be precursors of cysteine through the transsulfuration pathway, which is even more favored under oxidative conditions (Finkelstein, 1998; Mosharov et al., 2000). Therefore, our data suggest that glutathione is consumed to promote protection to cardiomyocytes after oxidative stress, as a defense mechanism against ROS.

The all-trans-4-oxoretinoic acid, a derivative of retinoic acid (which was used in the cell differentiation process), showed a slight fold change. Choudhary et al. (2008) showed that retinoic acid is a potential inhibitor of ROS generation, protecting cardiomyocytes from apoptosis generated by mechanical stress and Angiotensin II. In contrast, data about all-trans-4-oxoretinoic acid formation suggests that it inactivates growth and differentiation pathways (Pijnappel et al., 1993). Such results would account for the loss of the differentiated phenotype visualized in Figure 1 and Supplementary Figure S2B.

Another metabolite that was upregulated in cardiomyocytes under oxidative stress was Carnosine (1.48-fold). It is a dipeptide, composed of L-histidine and β-alanine amino acids, with roles in muscle cells regulating the calcium metabolism and protecting against ROS (Boldyrev et al., 2013). Carnosine functions as a chelant to sequestrate heavy metal ions. In cardiomyocytes, carnosine can buffer the intracellular medium during anaerobic glycolysis, protecting the cells from apoptosis by maintaining a constant acidity. Anaerobic glycolysis increases the production of lactic acid, which is dissociated in protons (H^+^) and lactate ions, and carnosine can sequester these protons, attenuating the acidosis (Baye et al., 2016; Zhao et al., 2019)**.** In hypertrophic and failing hearts, carnosine levels are depleted, suggesting its role against cardiac injury. Recent studies also indicate an interaction of carnosine with highly toxic lipid peroxidation products, such as 4-hydroxy-trans-2-nonenal and acrolein, which avoids damage and promotes cell protection in infarcted mouse hearts (Zhao et al., 2019). Therefore, the upregulation of carnosine may be protective for cardiomyocytes under OS, acting as a pro-survival metabolite.

Metabolites involved in the sphingolipid metabolism were mostly upregulated, such as sphingosine 1-phosphate (2.65-fold) and phytosphingosine (2.31-fold) in the recovery group. *In vivo* studies suggest that changes in sphingolipid metabolism may occur as a consequence of increased utilization or decreased synthesis on failing or hypertrophic hearts. For example, sphingosine can be used on ceramide synthesis, which is increased on failing or hypertrophic hearts. Furthermore, extensive changes on lipid metabolism were also associated with myocardial decompensation (Sansbury et al., 2014). Interestingly, sphingolipids can accumulate on tissues under conditions of cellular stress, causing the activation of some sphingolipid metabolizing enzymes, such as sphingomyelinases. Sphingosine 1-phosphate is a ceramide metabolite that can induce the generation of more oxygen reactive species and propagate inflammation (Nikolova-Karakashian and Reid, 2011). Studies also connect ceramides to heart failure, showing that it can lead to cardiomyocyte apoptosis by acting on the mitochondrial membranes, inducing ROS formation and promoting release of cytochrome c to initiate apoptosis. The upregulation of the sphingolipid metabolism demonstrated the persistence of the OS deleterious effects in the 48-h recovery cardiomyocytes.

## Conclusion

For the first time, data from a metabolomics study using differentiated H9c2 cardiomyocytes cultured *in vitro* were presented to analyze intracellular changes after oxidative stress, followed by cell recovery upon restoring initial culture medium composition. Our results show that the analysis of the intracellular metabolome by LC-MS and morphological assays can discriminate healthy cells from those suffering oxidative damage. This is an indication of a confident model to understand the effects of oxidative stress on the cardiovascular system and possible therapeutic targets. At the same time, the use of two LC-MS modes (RPLC and HILIC) with positive and negative ionization modes brought complementary information that could not be obtained using only one methodology, showing the importance of multiplatform analyses in untargeted metabolomics approaches.

## Data Availability

The datasets presented in this study can be found in online repositories. The names of the repository/repositories and accession number(s) can be found in the article/Supplementary Material.
