# Supplementary material for: Comprehending Cardiac Dysfunction by Oxidative Stress: Untargeted Metabolomics of In Vitro Samples
Source: Front Chem. 2022 Apr 8;10:836478. doi: 10.3389/fchem.2022.836478 (PMC9023746; doi:10.3389/fchem.2022.836478)
Supplement: Supplementary file 1 [file DataSheet1.PDF]

Supplementary Material

Comprehending Cardiac Dysfunction by Oxidative Stress: Untargeted Metabolomics of *in vitro* Samples

Alan Gonçalves Amaral[1], Alessandra Sussulini[2,3], Isabela Aparecida Moretto[4], André Alexandre de Thomaz[5], Flávia da Silva Zandonadi[2], Hans Rolando Zamora-Obando[1], Isabela Rocha[1], Regina Vincenzi Oliveira[6], Aline Mara dos Santos[4*], Ana Valéria Colnaghi Simionato[1,3*]

[1] Laboratory of Analysis of Biomolecules Tiselius, Institute of Chemistry, Department of Analytical Chemistry, State University of Campinas, Campinas, Brazil.

[2] Laboratory of Bioanalytics and Integrated Omics, Institute of Chemistry, Department of Analytical Chemistry, State University of Campinas, Campinas, Brazil.

[3] National Institute of Science and Technology for Bioanalytics – INCTBio, Institute of Chemistry, State University of Campinas, Campinas, Brazil.

[4] Institute of Biology, Department of Structural and Functional Biology, State University of Campinas, Campinas, Brazil.

[5] Institute of Physics "Gleb Wataghin", State University of Campinas, Campinas, Brazil

[6] Department of Chemistry, Federal University of São Carlos, São Carlos, Brazil.

*** Correspondence:**
Ana Valéria Colnaghi Simionato
avsimionato@unicamp.br

Aline Mara dos Santos
alinems@unicamp.br

Supplementary Tables

Table S1. Parameters used in each preprocessing step of mzXML files in MZmine 2.53 software.

Table S2. Tentative identification of potential biomarkers – OS vs. Control.

Table S3. Tentative identification of potential biomarkers – Recovery vs. Control.

Table S4. Tentative identification of potential biomarkers – Recovery vs. OS.

Supplementary Figures

Figure S1. A. MTT test to assess cell viability at concentrations of Ct (control), 50, 100, 400 and 600 µmol/L of H_2O_2 at 4, 8, 12 and 24 h exposure times. **** $p < 0.0001$ vs. control group. **B.** MTT assay to evaluate 24 and 48 h of cell recovery after 24 h of oxidative stress caused by cells exposure to 500 µmol/L of H_2O_2 for 24 h. Ct = control; ns = Not significant ($p \geq 0.05$).

Figure S2. A. Transmission images of H9c2 cardiomyoblasts before (Day 0) and every two days during the differentiation process (Day 2, 4 and 6) induced by reducing the percentage of FBS from 10% (v/v) to 1% (v/v) and supplementing with 10 nmol/L retinoic acid. **B.** Transmission images of H9c2 healthy cardiomyoblasts (Control - CT), with H_2O_2-induced oxidative stress for 24h (OS) and 24 h and 48 h of recovery (24h - recovery and 48h - recovery, respectively).

Figure S3. SR-SIM microscopy of cardiomyocytes from Control (CT), Oxidative Stress (OS) and 24 and 48 hours recovery groups. Magenta: actin; Yellow: paxillin; Blue: nucleus. Scale bar = 50 µm.

Figure S4. Summary of pathway analysis based on KEGG database. All the significantly match (*p* value < 0.05) were labeled. The color and size of each circle was based on *p* value and pathway impact value, respectively. Pathways that were significantly altered between (A) OS vs. Control, (B) Recovery vs. Control, and (C) Recovery vs. OS.

Figure S5. Boxplots of the features shown in the highlighted metabolic pathways (**A.** Alanine, Aspartate and Glutamate Metabolism; **B.** Anaerobic Glycolysis; **C.** Pyrimidine Biosynthesis and **D.** Glutathione Metabolism) and that showed biologically relevant change (**E**).

Figure S6. MS2 spectra acquired at 5.0 eV of L-aspartate ($[M-H]^-$) in HILIC negative ionization mode.

Figure S7. MS2 spectra acquired at 5.0 eV of L-asparagine ($[M-H]^-$) in HILIC negative ionization mode.

Figure S8. MS2 spectra acquired at 5.0 eV of glycerol 3-phosphate ($[M-H]^-$) in HILIC negative ionization mode.

Figure S9. MS2 spectra acquired at 5.0 eV of cytidine 5'-diphosphate (CDP) ($[M-H]^-$) in HILIC negative ionization mode.

Figure S10. MS2 spectra acquired at 5.0 eV of L-cystathionine ($[M-H]^-$) in HILIC negative ionization mode.

Figure S11. MS2 spectra acquired at 5.0 eV of taurine ($[M-H]^-$) in HILIC negative ionization mode.

Figure S12. MS2 spectra acquired at 5.0 eV of adenosine 5'-monophosphate (AMP) ($[M+H]^+$) in HILIC positive ionization mode.

Figure S13. MS2 spectra acquired at 5.0 eV of L-carnosine ($[M+Cl]^-$) in HILIC negative ionization mode.

Figure S14. A. MS2 spectra acquired at 5.0 eV of L-carnitine ($[M+H]^+$) in HILIC positive ionization mode.

Supplementary Tables

Table S1. Parameters used in each preprocessing step of mzXML files in MZmine 2.53 software.

Mass Detector (MS1)	Mass detector: centroid;<br>MS level: 1;<br>Noise level: 350 (RPLC-ESI(+)-MS); 300 (RPLC-ESI(-)-MS); 1000 (HILIC-ESI(+)-MS); 800 (HILIC-ESI(-)-MS)
Mass Detector (MS2)	Mass detector: centroid;<br>MS level: 2;<br>Noise level: 150 (RPLC-ESI(+)-MS); 100 (RPLC-ESI(-)-MS); 250 (HILIC-ESI(+)-MS); 200 (HILIC-ESI(-)-MS)
ADAP Chromatogram Builder	Min group size in # of scans: 3;<br>Group intensity threshold/min highest intensity: three times MS1 noise level;<br>*m/z* tolerance: 5.0 ppm.
Chromatogram Deconvolution	Algorithm: baseline cut-off;<br>*m/z* center calculation: median;<br>*m/z* range for MS2 scan pairing (Da): checked, 0.02;<br>RT range for MS2 scan pairing (min): checked, 0.1 min.
Isotopic Peaks Grouper	*m/z* tolerance: 5.0 ppm;<br>RT tolerance: 0.3 min (absolute);<br>Maximum charge: 2;<br>Representative isotope: most intense.
Alignment	*m/z* tolerance: 5.0 ppm;

	Weight for *m/z*: 75;<br>RT tolerance: 0.3 min (absolute);<br>Weight for RT: 25.
Filtering	Minimum peaks in a row: checked, 2;<br>*m/z*: 50-1300;<br>Keep only peaks with MS2 scan: checked.
Gap-Filling	Intensity tolerance: 10.0%;<br>*m/z* tolerance: 5.0 ppm;<br>RT tolerance: 0.3 min (absolute).

Table S2. Tentative identification of potential biomarkers – OS vs. Control.

t_R	*m/z*	*Adduct*	*Metabolite*	*Formula*	*p value*	*FDR*	*Fold change (log2)*
HILIC-MS-ESI							
Positive Mode							
3.063	176.0735	$[M+H-H_2O]^+$	N-Phenylacetylglycine	$C_{10}H_{11}NO_3$	0.00326	0.04100	2.5218
3.075	160.0480	$[M+H+Na]^{2+}$	DHAP(8:0)	$C_{11}H_{21}O_7P$	0.00307	0.04100	2.3281
7.542	318.3027	$[M+H]^+$	Phytosphingosine	$C_{18}H_{39}NO_3$	0.00090	0.01651	1.7586
12.140	210.0925	$[M+H-2H_2O]^+$	N-Phenylacetyl glutamic acid	$C_{14}H_{15}NO_3$	0.00053	0.01340	1.5720
12.497	291.1330	$[M+H]^+$	L-(N-Arginino)succinate	$C_{10}H_{18}N_4O_6$	0.00258	0.03988	-1.7604
13.008	445.0659	$[M+NH_4]^+$	Adenosine diphosphate (ADP)	$C_{10}H_{15}N_5O_{10}P_2$	0.00127	0.02130	1.5653

13.451	189.1592	$[M+H]^+$	7,8-Diaminononanoate	$C_9H_{20}N_2O_2$	0.00087	0.01651	1.6790
13.543	188.1164	$[M+2Na]^{2+}$	(7Z,10Z,13Z,16Z,19Z)-Docosapentaenoic acid (DPA)	$C_{22}H_{34}O_2$	0.00284	0.04071	0.9040
13.989	357.2515	$[M+H+NH_4]^{2+}$	PE(33:5)	$C_{38}H_{66}NO_8P$	0.00012	0.01024	2.0850
14.153	369.2510	$[M+NH_4]^+$	Sphingosine 1-phosphate	$C_{16}H_{34}NO_5P$	0.00069	0.01541	1.7930
Negative Mode							
0.164	520.9100	$[M+Na-2H]^-$	Inositol 1,3,4,5-tetraphosphate	$C_6H_{16}O_{18}P_4$	0.00206	0.02234	-0.2054
0.173	316.9478	$[M-H-H_2O]^-$	Myo-inositol 1,4-bisphosphate	$C_6H_{10}O_{12}P_2$	0.00717	0.04169	-0.1387
1.359	333.0919	$[M-H]^-$	Penicillin	$C_{16}H_{18}N_2O_4S$	0.00528	0.03663	3.5788
1.444	240.9791	$[M-2H]^{2-}$	Uridine 5'-triphosphate (UTP)	$C_9H_{15}N_2O_{15}P_3$	0.00867	0.04777	-0.7123
5.062	321.0233	$[M+FA-H]^-$	Gluconate 6-phosphate	$C_6H_{13}O_{10}P$	0.00223	0.02234	-1.7629
6.631	303.0798	$[M-H]^-$	N-Acetylaspartylglutamate	$C_{11}H_{16}N_2O_8$	0.00224	0.02234	1.6980
6.669	187.0360	$[M+FA-H]^-$	4-Imidazolone-5-acetate	$C_5H_6N_2O_3$	0.00265	0.02477	0.4029
9.048	402.0108	$[M-H]^-$	Cytidine 5'-diphosphate (CDP)	$C_9H_{15}N_3O_{11}P_2$	0.00026	0.01084	-3.4954
9.976	128.0352	$[M-H]^-$	4-Oxoproline	$C_5H_7NO_3$	0.00464	0.03452	1.1925
10.006	304.0019	$[M+Cl]^-$	N-Acetyl-L-glutamyl 5-phosphate	$C_7H_{12}NO_8P$	0.00094	0.01711	-0.2800
10.017	132.0302	$[M-H]^-$	L-Aspartate	$C_4H_7NO_4$	0.00127	0.01934	2.5562
10.193	242.0796	$[M-H]^-$<br>$[M+FA-H]^-$	Cytidine<br>N-Acetylhistidine	$C_9H_{13}N_3O_5$<br>$C_8H_{11}N_3O_3$	0.00429	0.03298	1.3544

10.456	552.9853	$[M+FA-H]^-$	Inosine 5'-triphosphate (ITP)	$C_{10}H_{15}N_4O_{14}P_3$	0.00140	0.01934	-1.8915
10.456	417.0107	$[M-2H]^{2-}$	Diadenosine tetraphosphate (Ap4A)	$C_{20}H_{28}N_{10}O_{19}P_4$	0.00356	0.02889	-1.3818
10.465	382.1018	$[M-H]^-$	Succinyladenosine	$C_{14}H_{17}N_5O_8$	0.00022	0.01048	2.3118
10.474	127.0499	$[M-H-H_2O]^-$	L-Glutamine	$C_5H_{10}N_2O_3$	0.00138	0.01934	1.5609
10.554	440.9968	$[M-H]^-$	Folic acid	$C_{19}H_{19}N_7O_6$	0.00047	0.01348	2.1153
10.571	104.0348	$[M-H]^-$	L-Serine	$C_3H_7NO_3$	0.00169	0.02074	0.8173
10.748	131.0451	$[M-H]^-$	L-Asparagine	$C_4H_8N_2O_3$	0.00613	0.03934	1.5638
10.782	402.9968	$[M-H]^-$	Uridine 5'-diphosphate (UDP)	$C_9H_{14}N_2O_{12}P_2$	0.00001	0.00316	-3.2898
12.760	243.0425	$[M-H]^-$	Penicillenic acid	$C_9H_2N_2O_4S$	0.00286	0.02503	1.7018
13.918	261.0737	$[M+Cl]^-$	Carnosine	$C_9H_{14}N_4O_3$	0.00042	0.01348	1.4808

Table S3. Tentative identification of potential biomarkers – Recovery vs. Control.

t_R	*m/z*	*Adduct*	*Metabolite*	*Formula*	*p value*	*FDR*	*Fold change (log2)*
RPLC-MS-ESI							
Positive Mode							
5.007	493.1366	$[M+H+NH_4]^{2+}$	(3E,5Z,8Z)-Tetradecatrienoyl-CoA	$C_{35}H_{52}N_7O_{17}P_3S$	7.46E-04	0.02344	0.7956
5.031	220.1150	$[M+2Na]^{2+}$	LysoPA(P-16:0/0:0)	$C_{19}H_{39}O_6P$	4.67E-03	0.04567	1.0894
18.998	427.2739	$[M+H+K]^{2+}$	PG(39:3)	$C_{45}H_{83}O_{10}P$	4.19E-03	0.04567	1.5007
Negative Mode							

1.152	920.2301	$[M-H]^-$	Decanoyl-CoA	$C_{31}H_{54}N_7O_{17}P_3S$	0.00030	0.00678	0.6422
1.302	318.0705	$[M+Cl]^-$	7-Methylinosine	$C_{11}H_{15}N_4O_5$	0.00275	0.02200	1.0096
1.543	147.0292	$[M+Na-2H]^-$	5-Aminoimidazole-4-carboxamide	$C_4H_6N_4O$	0.00088	0.01096	2.4836
5.008	437.2073	$[M-H-H_2O]^-$	LPA(20:5)	$C_{23}H_{37}O_7P$	0.00627	0.03514	2.0234
5.573	203.0789	$[2M-H]^-$	N-Formiminoglycine	$C_3H_6N_2O_2$	0.00285	0.02200	0.8576
6.725	784.1385	$[M-H]^-$	FAD	$C_{27}H_{33}N_9O_{15}P_2$	0.00012	0.00678	1.2061
7.144	455.0894	$[2M-H]^-$	L-Serine-phosphoethanolamine	$C_5H_{13}N_2O_6P$	0.00395	0.02584	1.2245

HILIC-MS-ESI

Positive Mode

2.480	184.0980	$[M+H]^+$	L-Adrenaline<br>L-Normetanephrine	$C_9H_{13}NO_3$	0.00251	0.02405	-0.9098
2.494	202.1107	$[M+NH_4]^+$	Hydronitroxide radical	$C_{12}H_{10}NO$	0.00108	0.01441	-0.7958
4.043	228.1122	$[M+H+Na]^{2+}$	LPA(18:3)	$C_{21}H_{37}O_7P$	0.00195	0.02242	-2.4121
7.542	318.3027	$[M+H]^+$	Phytosphingosine	$C_{18}H_{39}NO_3$	0.00011	0.00217	-2.3031
9.163	219.0335	$[M+2Na-H]^+$	Formiminoglutamic acid	$C_6H_{10}N_2O_4$	0.00336	0.02439	-0.6189
9.855	185.0344	$[M+K]^+$	L-Glutamine	$C_5H_{10}N_2O_3$	0.00539	0.03495	-0.2818
10.323	348.0739	$[M+H]^+$	Adenosine 5’-monophosphate (AMP)	$C_{10}H_{14}N_5O_7P$	0.00352	0.02439	3.6163
11.238	172.0024	$[M+K]^+$	L-Aspartate	$C_4H_7NO_4$	0.00564	0.03540	-0.6308
11.444	162.1125	$[M+H]^+$	L-Carnitine	$C_7H_{15}NO_3$	0.00236	0.02376	-1.9161
12.247	296.0690	$[M+K]^+$	L-α-Glycerylphosphoryl	$C_8H_{20}NO_6P$	0.00320	0.02439	-2.0447

			Choline				
12.550	399.1486	$[M+H]^+$<br>$[2M+Na]^+$	S-adenosyl-L-methionine (SAM)<br>N-Acetylglutamine	$C_{15}H_{22}N_6O_5S$<br>$C_7H_{12}N_2O_4$	0.00289	0.02439	-0.8168
13.008	445.0659	$[M+NH_4]^+$	Adenosine 5'-diphosphate (ADP)<br>2'-Deoxyguanosine 5'-diphosphate (dGDP)	$C_{10}H_{15}N_5O_{10}P_2$	0.00005	0.00177	-1.6037
13.025	423.0828	$[M+K]^+$	S-Adenosyl-L-homocysteine	$C_{14}H_{20}N_6O_5S$	0.00063	0.00967	-2.2944
13.051	136.0512	$[M+H-H_2O]^+$	N-Dimethyl-2-aminoethylphosphonate	$C_4H_{12}NO_3P$	0.00278	0.02439	-1.2734
13.543	188.1164	$[M+2Na]^{2+}$	(7Z,10Z,13Z,16Z,19Z)-Docosapentaenoic acid (DPA)	$C_{22}H_{34}O_2$	0.00028	0.00505	-1.5263
13.989	357.2515	$[M+H+NH_4]^{2+}$	PE(33:5)	$C_{38}H_{66}NO_8P$	0.00002	0.00113	-2.6148
14.153	369.2511	$[M+NH_4]^+$	Sphingosine 1-phosphate	$C_{16}H_{34}NO_5P$	0.00009	0.00209	-2.6476
Negative Mode							
1.444	240.9791	$[M-2H]^{2-}$	Uridine 5'-triphosphate (UTP)	$C_9H_{15}N_2O_{15}P_3$	0.00679	0.02835	0.4362
3.408	558.3123	$[M+Na-2H]^-$	PC(18:0)	$C_{26}H_{52}NO_8P$	0.00031	0.00503	-1.0113
5.312	231.9998	$[M+K-2H]^-$	Dopaquinone	$C_9H_9NO_4$	0.00001	0.00067	-1.8674
5.314	259.0188	$[M-H]^-$	α-D-Glucose 6-phosphate<br>β-D-Glucose 6-phosphate	$C_6H_{13}O_9P$	0.00003	0.00123	-2.0195
5.643	177.0403	$[M-H-H_2O]^-$	Gluconic acid	$C_6H_{12}O_7$	0.00021	0.00397	-2.4539
5.842	135.0303	$[M+FA-H]^-$	L-Lactate	$C_3H_6O_3$	0.00300	0.01654	-1.5921

6.392	173.0076	$[M-H-H_2O]^-$	Citrate	$C_6H_8O_7$	0.01056	0.03876	-0.4855
6.631	303.0798	$[M-H]^-$	N-Acetylaspartylglutamate	$C_{11}H_{16}N_2O_8$	0.00006	0.00186	-2.4378
6.669	187.0360	$[M+FA-H]^-$	4-Imidazolone-5-acetate	$C_5H_6N_2O_3$	0.00035	0.00503	-1.0829
6.947	419.7714	$[M-2H]^{2-}$	PE(44:9)	$C_{49}H_{80}NO_8P$	0.00573	0.02550	2.0699
6.970	166.0627	$[M+Cl]^-$	L-Leucine	$C_6H_{13}NO_2$	0.00581	0.02550	0.5633
8.408	124.0075	$[M-H]^-$	Taurine	$C_2H_7NO_3S$	0.00243	0.01430	-0.7275
8.810	171.0054	$[M-H]^-$	Glycerol 3-phosphate	$C_3H_9O_6P$	0.00079	0.00890	-0.8404
9.048	402.0108	$[M-H]^-$	Cytidine 5'-diphosphate (CDP)	$C_9H_{15}N_3O_{11}P_2$	0.00175	0.01164	4.5446
9.397	156.0276	$[M-H-H_2O]^-$	N-Acetyl-L-aspartic acid	$C_6H_9NO_5$	0.00057	0.00710	-1.4248
9.400	292.0021	$[M+FA-H]^-$	N-Phosphotaurocyamine	$C_3H_{10}N_3O_6PS$	0.00035	0.00503	-1.0064
9.976	128.0352	$[M-H-H_2O]^-$	L-Glutamate	$C_5H_9NO_4$	0.01074	0.03876	-1.0396
9.999	315.0803	$[M-H-H_2O]^-$	Penicillin G	$C_{16}H_{18}N_2O_4S$	0.00621	0.02697	-0.9066
10.006	304.0019	$[M+Cl]^-$	N-Acetyl-L-glutamate 5-phosphate	$C_7H_{12}NO_8P$	0.00115	0.01041	0.3379
10.017	132.0302	$[M-H]^-$<br>$[M+FA-H]^-$	L-Aspartate<br>Dehydroalanine	$C_4H_7NO_4$<br>$C_3H_5NO_2$	0.00426	0.02084	-2.3881
10.033	221.9993	$[M-H]^-$	4-Methyl-5-(2-phosphooxyethyl)thiazole	$C_6H_{10}NO_4PS$	0.00534	0.02491	-0.7275
10.144	306.0762	$[M-H]^-$	Glutathione	$C_{10}H_{17}N_3O_6S$	0.00071	0.00830	-1.1032
10.164	225.0613	$[M+FA-H]^-$	Myo-Inositol	$C_6H_{12}O_6$	0.01097	0.03899	-0.5220
10.193	242.0796	$[M-H]^-$	Cytidine	$C_9H_{13}N_3O_5$	0.00098	0.00999	-1.8664
10.465	382.1018	$[M-H]^-$	Succinyladenosine	$C_{14}H_{17}N_5O_8$	0.00402	0.02023	-1.7590

10.474	127.0499	$[M-H-H_2O]^-$	L-Glutamine	$C_5H_{10}N_2O_3$	0.00405	0.02023	-1.0901
10.554	440.1374	$[M-H]^-$	Folic acid	$C_{19}H_{19}N_7O_6$	0.00179	0.01164	-1.3984
10.560	272.0876	$[M+FA-H]^-$	Deoxycytidine	$C_9H_{13}N_3O_4$	0.00105	0.01000	-1.2317
10.571	104.0348	$[M-H]^-$	L-Serine	$C_3H_7NO_3$	0.00372	0.01999	-0.5142
10.748	131.0451	$[M-H]^-$	L-Asparagine	$C_4H_8N_2O_3$	0.00122	0.01048	-1.7184
10.751	294.0516	$[M+K-2H]^-$	L-α-Glycerylphosphoryl choline	$C_8H_{20}NO_6P$	0.00058	0.00710	-2.1310
10.772	335.0080	$[M+Cl]^-$	Cinnavalininate	$C_{14}H_8N_2O_6$	0.00164	0.01164	1.4945
10.782	402.9968	$[M-H]^-$	Uridine 5'-diphosphate (UDP)	$C_9H_{14}N_2O_{12}P_2$	1.63E-07	0.00007	2.4717
11.532	247.0506	$[M-H]^-$	Pyridoxamine 5'-phosphate	$C_8H_{13}N_2O_5P$	0.00686	0.02835	-1.1560
12.760	243.0425	$[M-H]^-$	Penicillenic acid	$C_9H_{12}N_2O_4S$	0.00006	0.00186	-2.5858
13.234	212.0199	$[M+FA-H]^-$	Quinolinic acid	$C_7H_5NO_4$	0.01311	0.04511	0.2659
13.861	293.0814	$[M+Cl]^-$	Glycerophosphocholine	$C_8H_{21}NO_6P$	0.00972	0.03666	-2.7024
14.357	342.0882	$[M+FA-H]^-$	5'-Methylthioadenosine	$C_{11}H_{15}N_5O_3S$	0.00120	0.01048	-1.1327

Table S4. Tentative identification of potential biomarkers – Recovery vs. OS.

t_R	***m/z***	***Adduct***	***Metabolite***	***Formula***	***p value***	***FDR***	***Fold change (log2)***
HILIC-MS-ESI							
Positive Mode							
3.072	357.0910	$[2M+H]^+$	L-Cysteinylglycine	$C_5H_{10}N_2O_3S$	0.00004	0.00379	1.6326

3.075	160.0480	$[M+H+NH_4]^{2+}$	N-Acetyl-glucosamine 1-phosphate<br><br>N-Acetyl-D-glucosamine 6-phosphate	$C_8H_{16}NO_9P$	0.00006	0.00379	1.7962
4.028	163.0628	$[M+H+NH_4]^{2+}$	Glutathione	$C_{10}H_{17}N_3O_6S$	0.00299	0.03759	0.9091
4.043	228.1122	$[M+H+NH_4]^{2+}$	LPA(18:3)	$C_{21}H_{37}O_7P$	0.00198	0.03076	-1.5707
10.665	84.0447	$[M+H+NH_4]^{2+}$	2-Formylamino<br><br>benzaldehyde	$C_8H_7NO_2$	0.00206	0.03076	0.4577
11.444	162.1125	$[M+H]^+$	L-Carnitine	$C_7H_{15}NO_3$	0.00008	0.00379	-2.1290
11.748	169.0925	$[M+H+Na]^{2+}$	All-trans-4-oxoretinoic acid	$C_{20}H_{26}O_3$	0.00081	0.02203	0.9436
14.153	369.2511	$[M+NH_4]^+$	Sphingosine 1-phosphate	$C_{16}H_{34}NO_5P$	0.00172	0.03076	-0.8546
Negative Mode							
1.359	333.0919	$[M-H]^-$	Penicillin	$C_{16}H_{18}N_2O_4S$	0.00023	0.01618	2.3434
3.714	295.0353	$[M-H-H_2O]^-$	5′-Phosphoribosyl-N-formylglyciamide	$C_8H_{15}N_2O_9P$	0.00067	0.01799	1.2439
3.738	128.0354	$[M-H]^-$	5-Oxoproline	$C_5H_7NO_3$	0.00112	0.02017	0.7507
5.643	177.0403	$[M+FA-H]^-$	Glutaric acid	$C_5H_8O_4$	0.00360	0.03973	-0.9618
6.633	285.0725	$[2M-H]^-$	5-(2-Hydroxy)-4-methylthiazole	C_6H_9NOS	0.00337	0.03919	-0.7244
6.669	187.0360	$[M+FA-H]^-$	4-Imidazolone-5-acetate	$C_5H_6N_2O_3$	0.00108	0.02017	-0.6799
9.397	156.0276	$[M+Na-2H]^-$	4-Hydroxy-L-threonine	$C_4H_9NO_4$	0.00165	0.02375	-0.9481
9.400	292.0021	$[M+FA-H]^-$	N-Phosphotaurocyamine	$C_3H_{10}N_3O_6PS$	0.00064	0.01799	-0.7458
9.414	495.9643	$[M+K-2H]^-$	8-Oxoguanosine 5′-phosphate	$C_{10}H_{15}N_5O_{12}P_2$	0.00084	0.01905	-0.5410

9.898	142.0126	$[M-H-H_2O]^-$	N-Formyl-L-aspartate	$C_5H_7NO_5$	0.00500	0.04885	-0.3827
10.571	104.0348	$[M-H]^-$	L-Serine	$C_3H_7NO_3$	0.00437	0.04471	0.3031
10.782	402.9968	$[M-H]^-$	Uridine 5’-diphosphate (UDP)	$C_9H_{14}N_2O_{12}P_2$	0.00112	0.02017	-0.8181
12.751	221.0602	$[M-H]^-$	L-Cystathionine	$C_7H_{14}N_2O_4S$	0.00038	0.01799	-1.3566
14.357	342.0882	$[M+FA-H]^-$	5’-Methylthioadenosine	$C_{11}H_{15}N_5O_3S$	0.00263	0.03647	-1.0488

Supplementary Figures

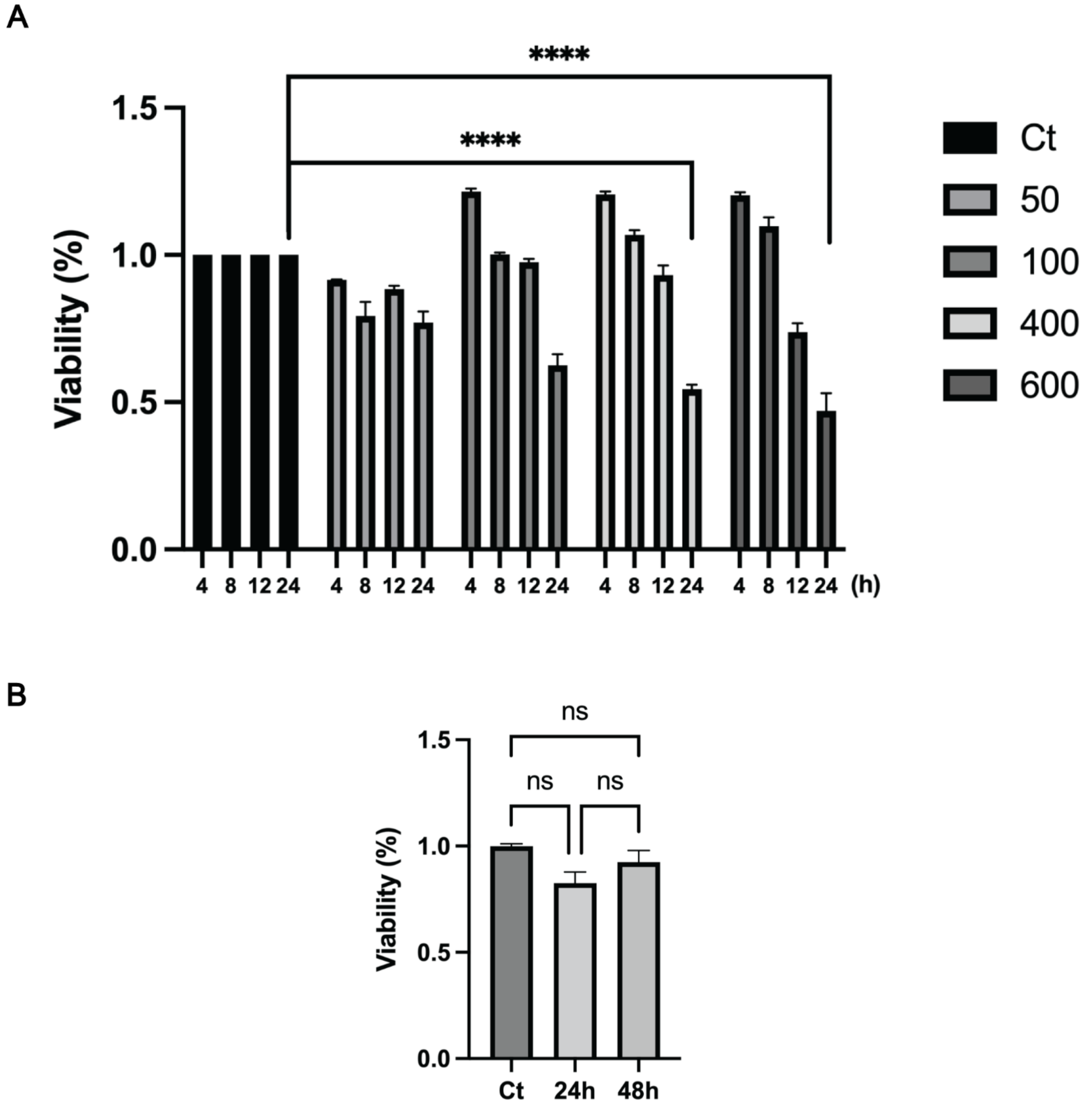

Figure S1. A. MTT test to assess cell viability at concentrations of Ct (control), 50, 100, 400 and 600 µmol/L of H_2O_2 at 4, 8, 12 and 24 h exposure times. **** $p < 0.0001$ vs. control group. **B.** MTT assay to evaluate 24 and 48 h of cell recovery after 24 h of oxidative stress caused by cells exposure to 500 µmol/L of H_2O_2 for 24 h. Ct = control; ns = Not significant ($p \geq 0.05$).

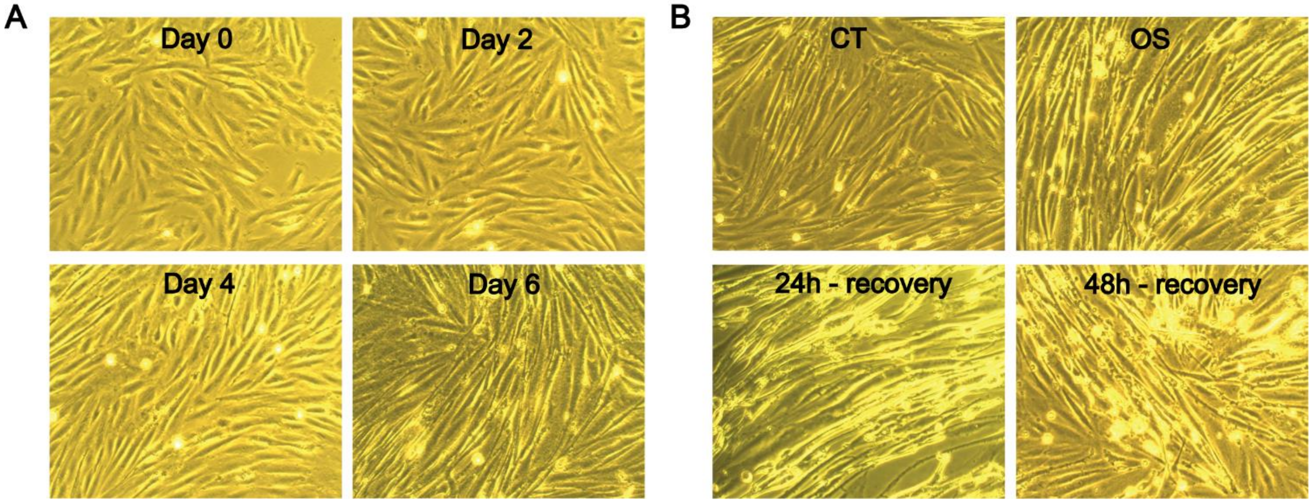

Figure S2. A. Transmission images of H9c2 cardiomyoblasts before (Day 0) and every two days during the differentiation process (Day 2, 4 and 6) induced by reducing the percentage of FBS from 10% (v/v) to 1% (v/v) and supplementing with 10 nmol/L retinoic acid. **B.** Transmission images of H9c2 healthy cardiomyoblasts (Control - CT), with H_2O_2-induced oxidative stress for 24h (OS) and 24 h and 48 h of recovery (24h - recovery and 48h - recovery, respectively).

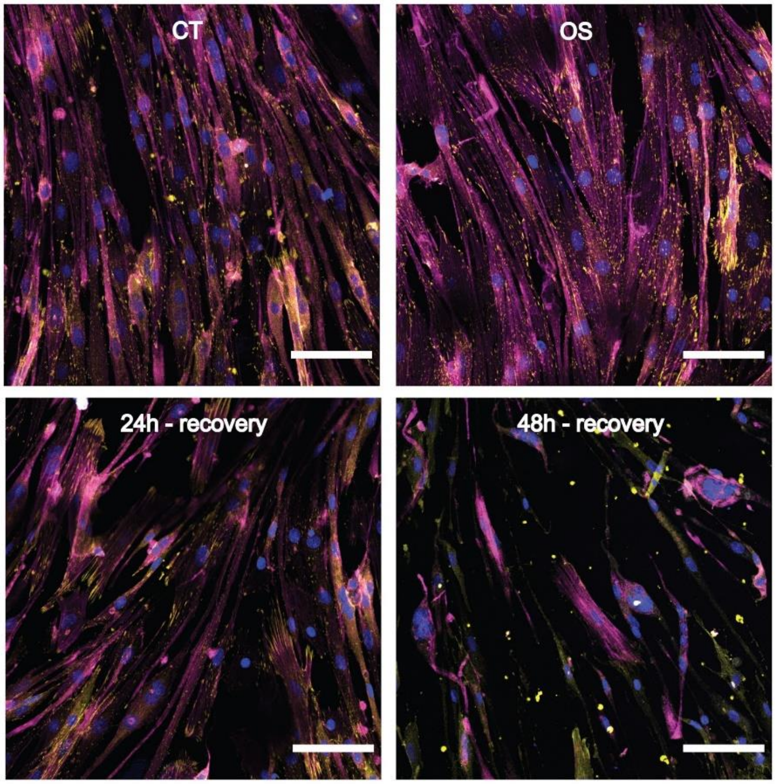

Figure S3. SR-SIM microscopy of cardiomyocytes from Control (CT), Oxidative Stress (OS) and 24 and 48 hours recovery groups. Magenta: actin; Yellow: paxillin; Blue: nucleus. Scale bar = 50 µm.

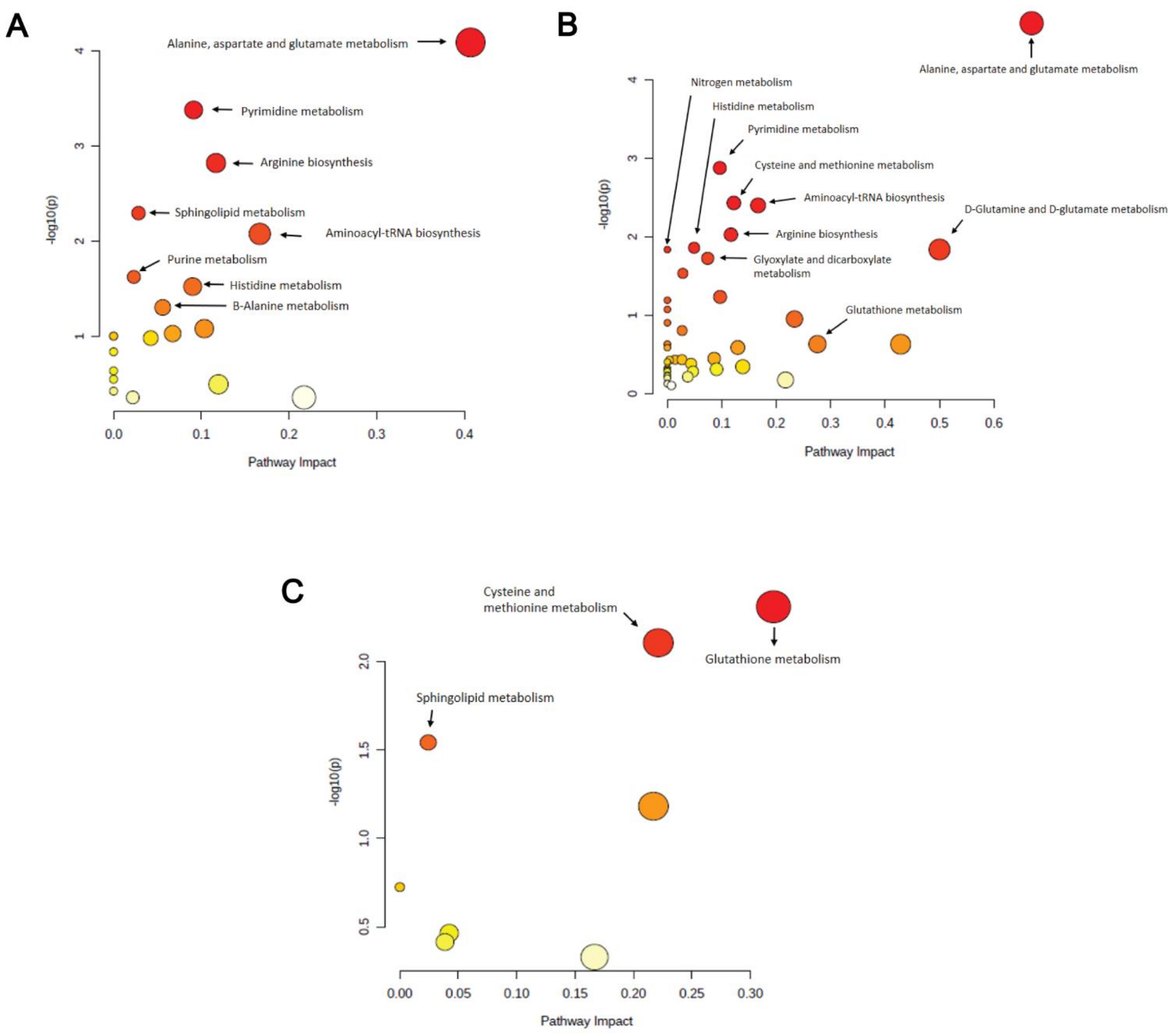

Figure S4. Summary of pathway analysis based on KEGG database. All the significantly match (p value < 0.05) were labeled. The color and size of each circle was based on p value and pathway impact value, respectively. Pathways that were significantly altered between (A) OS vs. Control, (B) Recovery vs. Control, and (C) Recovery vs. OS.

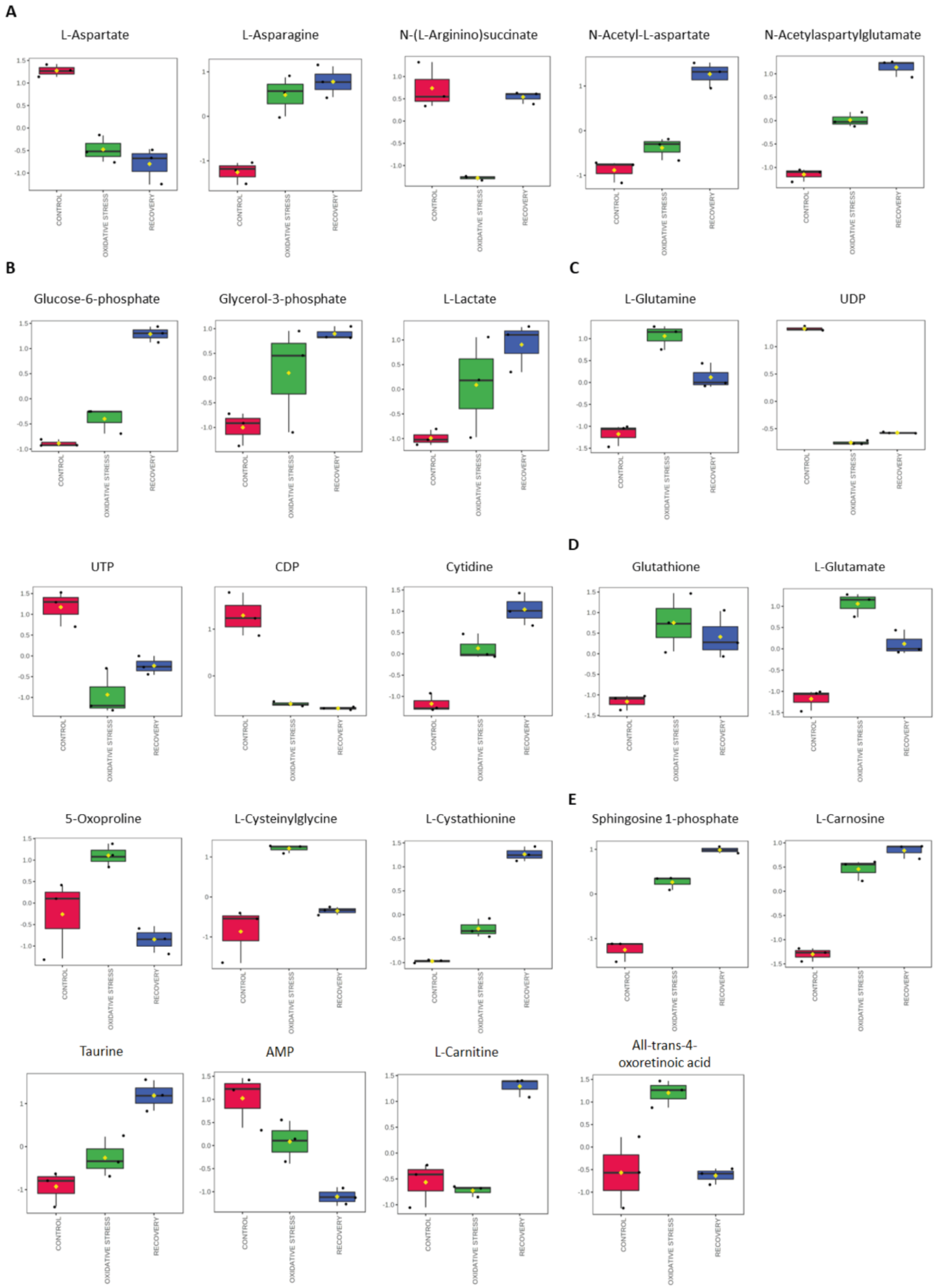
A
L-Aspartate
L-Asparagine
N-(L-Arginino)succinate
N-Acetyl-L-aspartate
N-Acetylaspartylglutamate
B
Glucose-6-phosphate
Glycerol-3-phosphate
L-Lactate
C
L-Glutamine
UDP
UTP
CDP
Cytidine
D
Glutathione
L-Glutamate
5-Oxoproline
L-Cysteinylglycine
L-Cystathionine
E
Sphingosine 1-phosphate
L-Carnosine
Taurine
AMP
L-Carnitine
All-trans-4-oxoretinoic acid
CONTROL
OXIDATIVE STRESS
RECOVERY

Figure S5. Boxplots of the features shown in the highlighted metabolic pathways (**A.** Alanine, Aspartate and Glutamate Metabolism; **B.** Anaerobic Glycolysis; **C.** Pyrimidine Biosynthesis and **D.** Glutathione Metabolism) and that showed biologically relevant change (**E**).

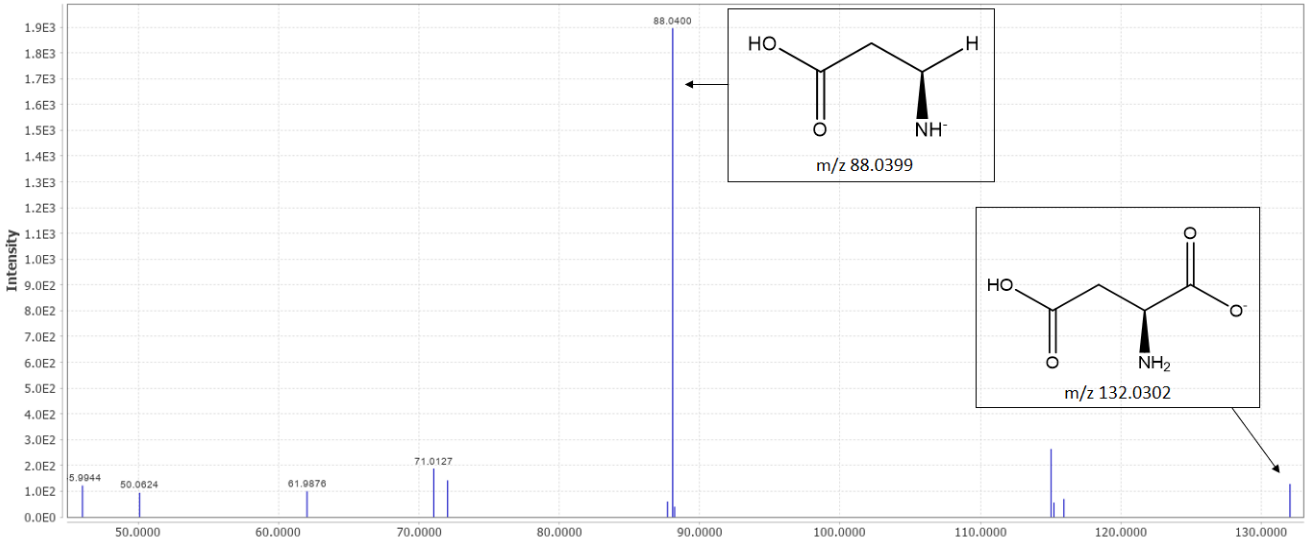

Figure S6. MS2 spectra acquired at 5.0 eV of L-aspartate ($[M-H]^-$) in HILIC negative ionization mode.

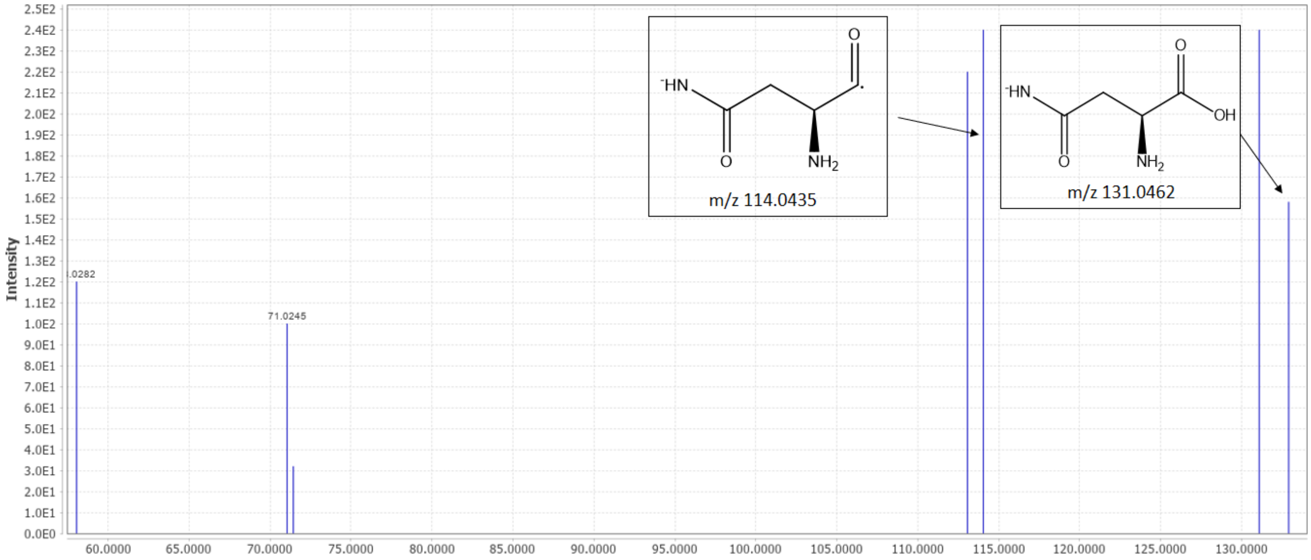

Figure S7. MS2 spectra acquired at 5.0 eV of L-asparagine ($[M-H]^-$) in HILIC negative ionization mode.

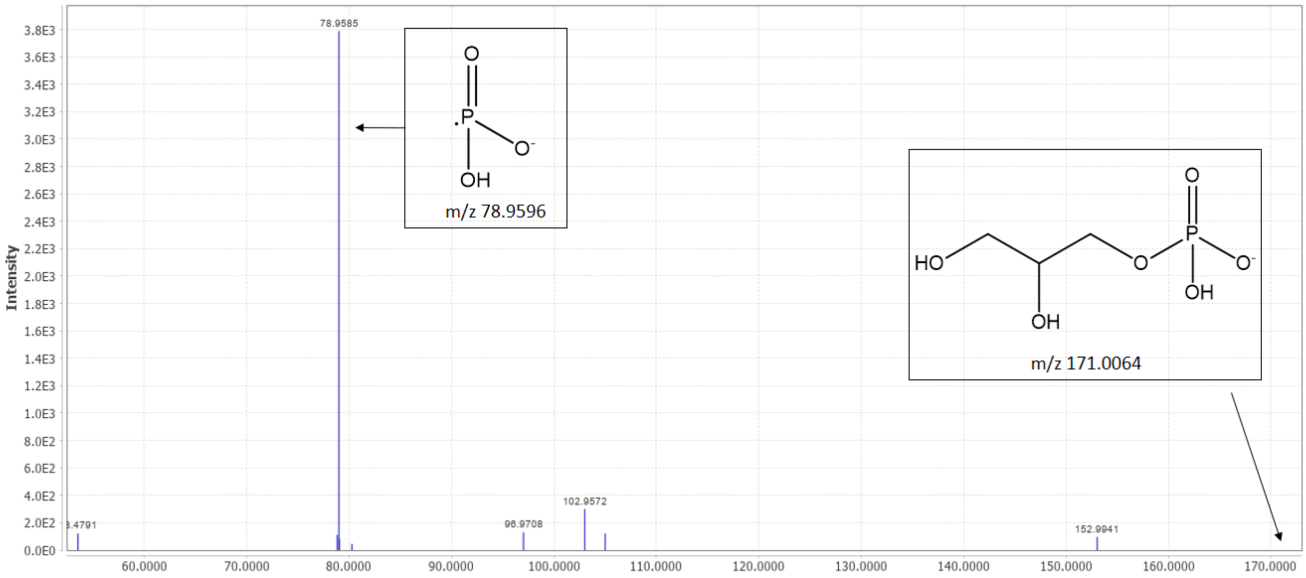

Figure S8. MS2 spectra acquired at 5.0 eV of glycerol 3-phosphate ($[M-H]^-$) in HILIC negative ionization mode.

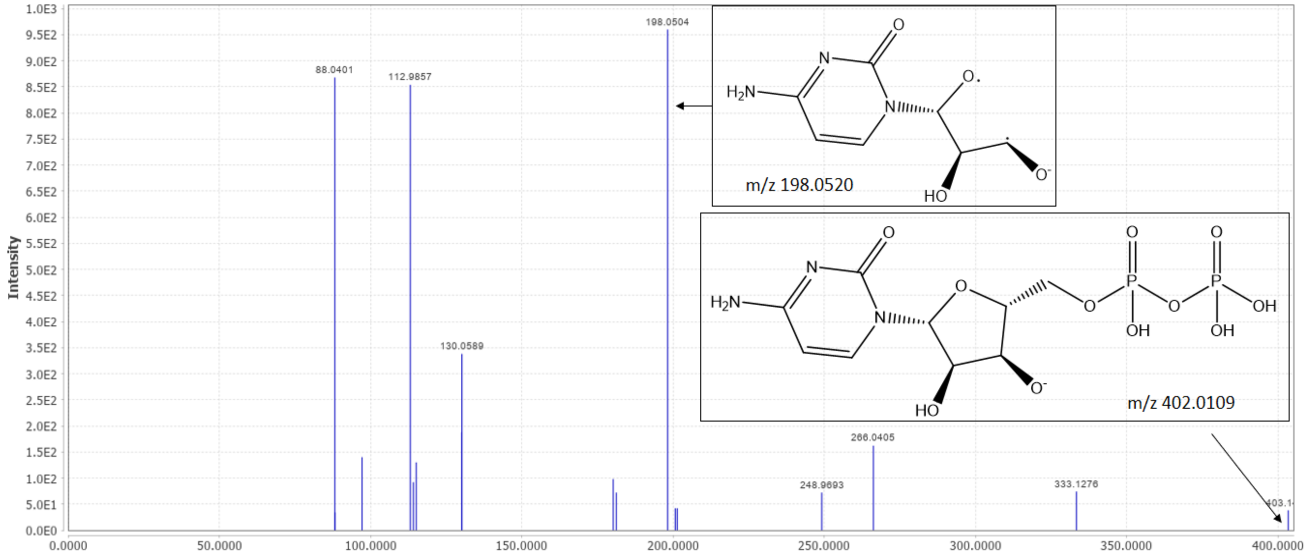

Figure S9. MS2 spectra acquired at 5.0 eV of cytidine 5’-diphosphate (CDP) ($[M-H]^-$) in HILIC negative ionization mode.

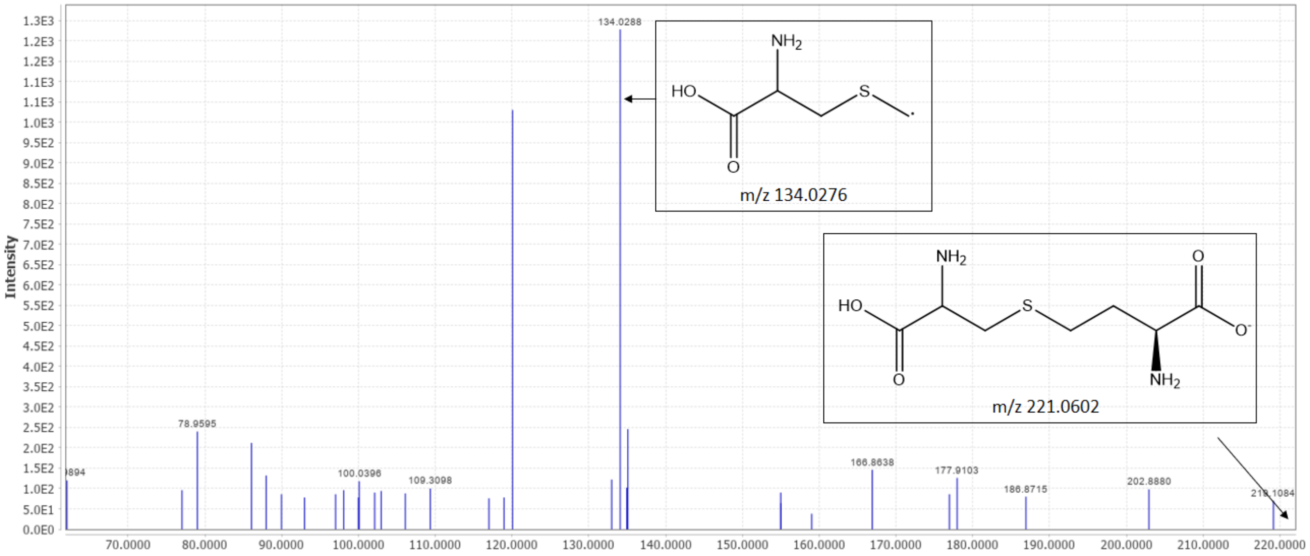

Figure S10. MS2 spectra acquired at 5.0 eV of L-cystathionine ($[M-H]^-$) in HILIC negative ionization mode.

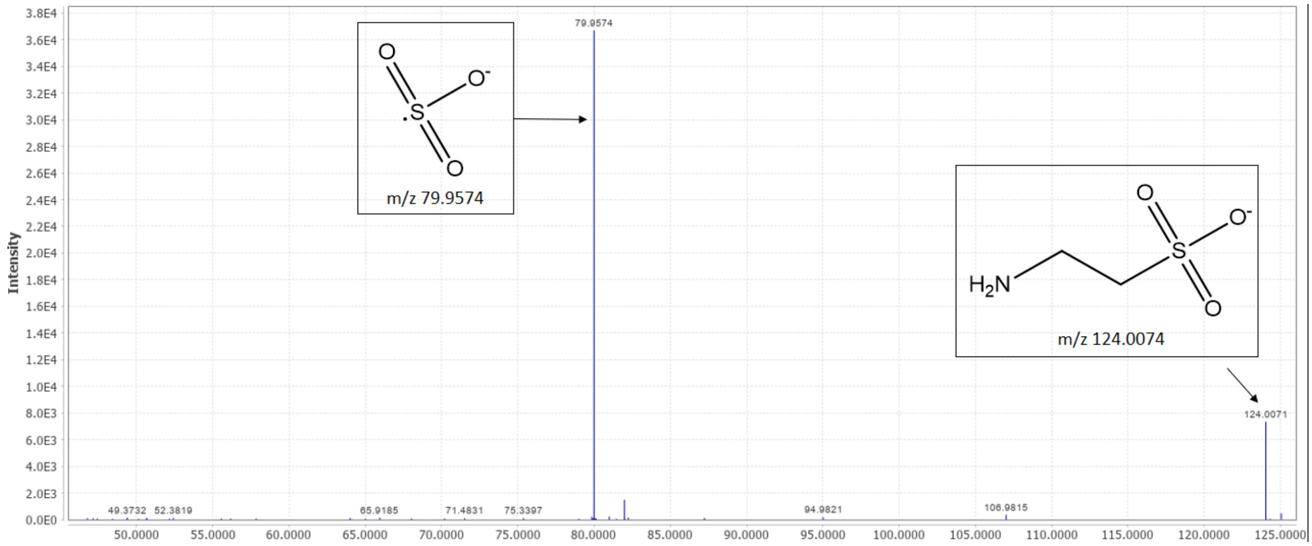

Figure S11. MS2 spectra acquired at 5.0 eV of taurine ($[M-H]^-$) in HILIC negative ionization mode.

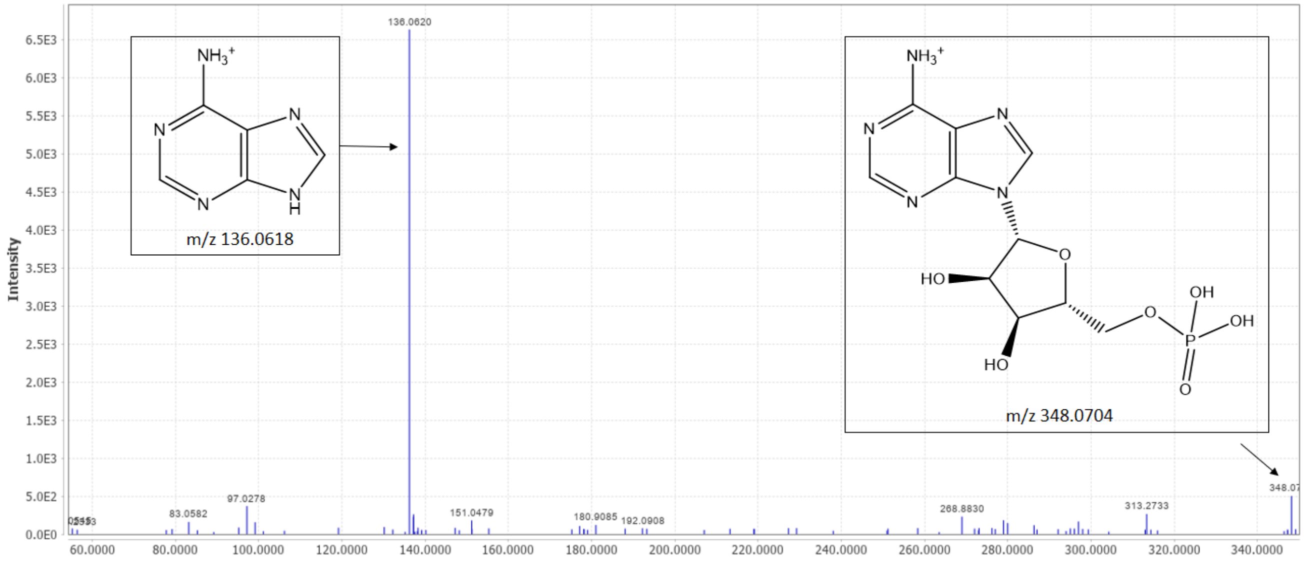

Figure S12. MS2 spectra acquired at 5.0 eV of adenosine 5’-monophosphate (AMP) ([M+H]+) in HILIC positive ionization mode.

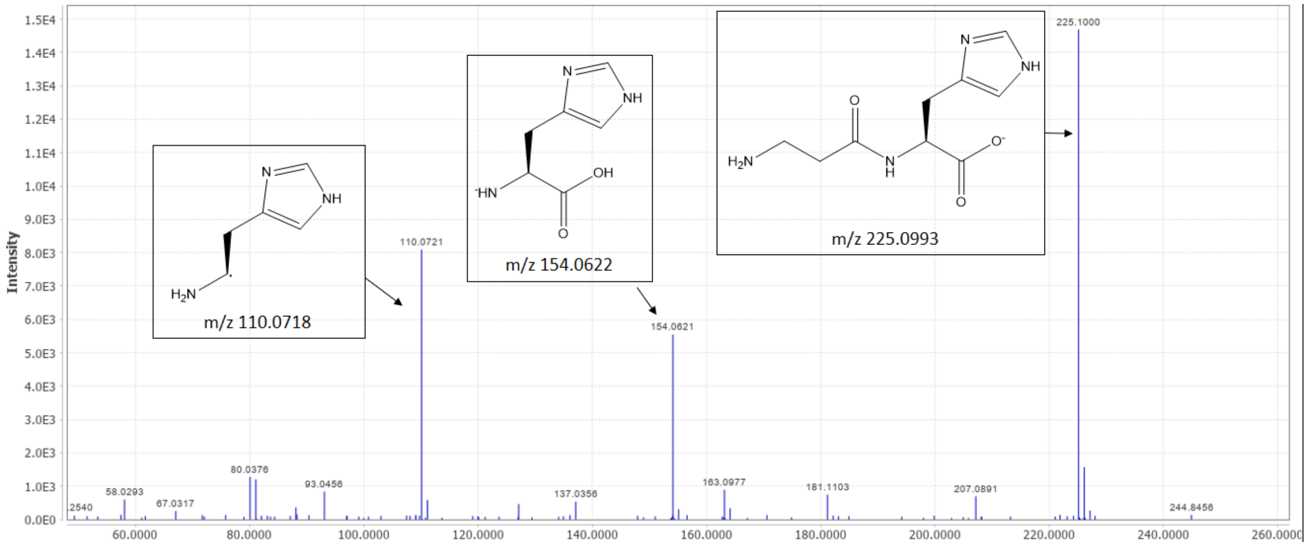

Figure S13. MS2 spectra acquired at 5.0 eV of L-carnosine ([M+Cl]-) in HILIC negative ionization mode.

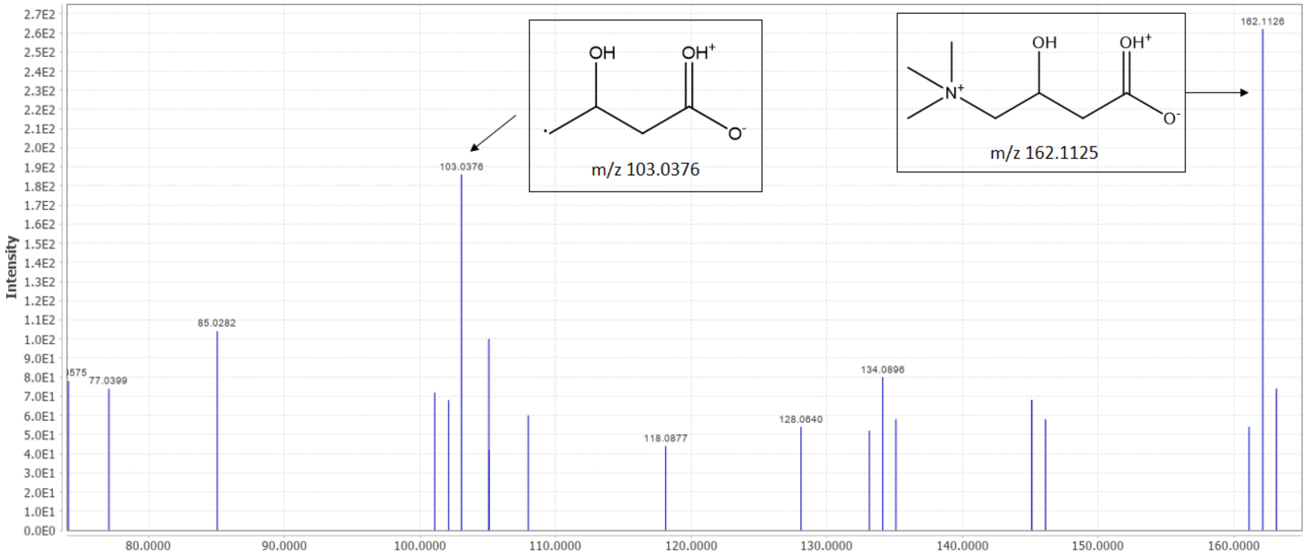

Figure S14. A. MS2 spectra acquired at 5.0 eV of L-carnitine ([M+H]+) in HILIC positive ionization mode.
